# Comprehensive *in silico* survey of the *Mycolicibacterium* mobilome reveals an as yet underexplored diversity

**DOI:** 10.1099/mgen.0.000533

**Published:** 2021-02-23

**Authors:** Sergio Mascarenhas Morgado, Ana Carolina Paulo Vicente

**Affiliations:** ^1^​ Laboratory of Molecular Genetics of Microorganisms, Oswaldo Cruz Institute, Rio de Janeiro, Brazil

**Keywords:** AICE, antibiotic and metal resistance, *Mycobacterium*, plasmid, prophage, T7SS-PE/PPE

## Abstract

The mobilome plays a crucial role in bacterial adaptation and is therefore a starting point to understand and establish the gene flow occurring in the process of bacterial evolution. This is even more so if we consider that the mobilome of environmental bacteria can be the reservoir of genes that may later appear in the clinic. Recently, new genera have been proposed in the family *Mycobacteriaceae *, including the genus *Mycolicibacterium *, which encompasses dozens of species of agricultural, biotechnological, clinical and ecological importance, being ubiquitous in several environments. The current scenario in the *Mycobacteriaceae* mobilome has some bias because most of the characterized mycobacteriophages were isolated using a single host strain, and the few plasmids reported mainly relate to the genus *Mycobacterium *. To fill in the gaps in these issues, we performed a systematic *in silico* study of these mobile elements based on 242 available genomes of the genus *Mycolicibacterium *. The analyses identified 156 putative plasmids (19 conjugative, 45 mobilizable and 92 non-mobilizable) and 566 prophages in 86 and 229 genomes, respectively. Moreover, a contig was characterized by resembling an actinomycete integrative and conjugative element (AICE). Within this diversity of mobile genetic elements, there is a pool of genes associated with several canonical functions, in addition to adaptive traits, such as virulence and resistance to antibiotics and metals (mercury and arsenic). The type-VII secretion system was a common feature in the predicted plasmids, being associated with genes encoding virulent proteins (EsxA, EsxB, PE and PPE). In addition to the characterization of plasmids and prophages of the family *Mycobacteriaceae *, this study showed an abundance of these genetic elements in a dozen species of the genus *Mycolicibacterium *.

## Data Summary

The genomic data analysed in this work are listed in Tables S1 and S3 of Supplementary File 1 (available in the online version of this article). All supplementary files can be found on Figshare at 10.6084/m9.figshare.13286357

**Impact Statement**

Mobile genetic elements, such as plasmids and bacteriophages, are key players in the adaptation and evolution of bacteria. The family *Mycobacteriaceae *, which includes several genera with species impacting the environment and the clinic, remains largely underexplored with regard to its mobilome. Studies based on *in silico* analyses have revealed scenarios that can be explored in depth and focused on specific issues. Using this approach and based on hundreds of *Mycolicibacterium* genomes, we were able to show an abundance of plasmids and prophages in several species of the genus. Besides, a rare actinomycete integrative and conjugative element has been identified in *Mycolicibacterium novocastrense *. Therefore, this study provides evidence that expands the landscape of the *Mycobacteriaceae* mobilome, revealing a distribution and diversity of these elements within the genus *Mycolicibacterium *.

## Introduction

The family *Mycobacteriaceae* is composed of hundreds of species of agricultural, biotechnological, clinical and ecological importance, being ubiquitous in several environments. Recently, one of its genera, *Mycobacterium,* was reclassified into an emended genus *Mycobacterium* and four new genera. Most species in the family *Mycobacteriaceae* belong to the genera *Mycobacterium* (slowly growing mycobacteria), which includes several human pathogens, and *Mycolicibacterium* (rapidly growing mycobacteria), saprophyte organisms that mainly comprise environmental species and opportunistic pathogens [1]. Although the family *Mycobacteriaceae* has a huge diversity of species, its mobilome is underexplored [2].

Mobile genetic elements, which collectively constitute the bacterial mobilome, are the main vectors of horizontal gene transfer in these organisms. They represent a source of diversity that can confer novel traits and play a role in the ecology and evolution of bacteria [3–6]. The study of these elements is the starting point for understanding the dynamics of gene flow within a bacterial community and how it affects its diversity and density [7]. These genetic entities include DNA elements such as integrons, plasmids, integrative conjugative elements, insertion sequences, bacteriophages and transposons that can mobilize and integrate into the host genome [2, 8].

So far, plasmids, a mobilome central element, have been considered to be rare in the family *Mycobacteriaceae* [9, 10]. Despite this, plasmids have been associated with the evolution and dissemination of the major specialized secretion system among hundreds of mycobacteria species, namely the type-VII secretion system (T7SS or ESX). This secretion system is encoded by six paralogous loci (ESX-1, -2, -3, -4, -4-*bis* and -5) in plasmids and chromosomes [11–13]. To date, most of the plasmids identified in this family (*n*=94) belong to the genus *Mycobacterium* (https://www.ncbi.nlm.nih.gov/genome/browse#!/plasmids/mycobacterium), probably due to its clinical relevance. Regarding the genus *Mycolicibacterium *, despite its 86 described species, there is only one report characterizing two of the 19 plasmids identified so far in this genus of bacteria [14] (https://www.ncbi.nlm.nih.gov/genome/browse#!/plasmids/mycolicibacterium). Additionally, in the NCBI database, there is a bias as only genomes of defined species are associated with plasmids. Moreover, despite several reports of *Mycolicibacterium* genomes [15–26], these studies rarely survey plasmids among their contigs. Thus, only a few plasmids have been reported in this genus, mainly from unrecognized genomes [2, 14, 27–30]. Despite the reclassification of *Mycobacterium *, some *Mycobacterium* sp. genomes that are in fact *Mycolicibacterium* remain named as *Mycobacterium* (https://www.ncbi.nlm.nih.gov/genome/browse/#!/prokaryotes/13563/) (e.g. *Mycobacterium* sp. JS623, *Mycobacterium* sp. KMS and *Mycobacterium* sp. YC-RL4, as observed in the phylogeny of Morgado and Vicente, 2020), some of them presenting plasmids.

In contrast to plasmids, there are thousands of recognized mycobacteriophages (http://phagesdb.org), which were classified in dozens of clusters based on their gene content [31]. Most of these mycobacteriophages were from the environment and isolated using a single host strain: *Mycolicibacterium smegmatis* mc^2^155 [31–33]. Thus, the range of *Mycolicibacterium* species that are infected by bacteriophages is unknown, and knowledge of the diversity of these mobile genetic elements in other species is limited. Therefore, the mobilome of *Mycolicibacterium* species has a great potential for an abundance and diversity of bacteriophages yet to be revealed.

Due to the relevance of mobile genetic elements in the adaptation and evolution of bacteria, here we performed a comprehensive *in silico* survey, focusing on plasmids and prophages, based on all 242 complete and draft *Mycolicibacterium* genomes available at NCBI. Following our workflow, analyses revealed a total of 156 plasmids within 86 genomes, distributed in several *Mycolicibacterium* species. These plasmids were mainly predicted to be non-mobilizable and their cargo genes presented a prevalence of T7SS and resistance genes (metal and antibiotics) with low identity to clinical sequences. In addition, one of the predicted plasmids may be an integrative and conjugative element of actinomycetes (AICE). Among the prophages, 566 were predicted in 229/242 genomes, being distributed in 94 % of the species, but only 40 of the prophages were assigned as intact. They carried more antibiotic resistance genes than plasmids, but also with low sequence identity. Among the antibiotic resistance genes identified in the *Mycolicibacterium* mobilome, there was a prophage encoding an Arr protein presenting 92 % identity to the Arr protein encoded in *M. smegmatis *. Most of the intact prophages identified could not be assigned to recognized mycobacteriophage clusters, suggesting an unrevealed diversity among species of the genus *Mycolicibacterium *.

## Methods

### Genome sequences analysed

A total of 242 *Mycolicibacterium* genomes were retrieved from the NCBI ftp site (ftp://ftp.ncbi.nlm.nih.gov/genomes/genbank/bacteria) in January 2020, encompassing 69 species and 27 sp. genomes (Table S1). Representative mycobacteriophage genomes (*n*=193; Table S2) were obtained from the Actinobacteriophage Database (http://phagesdb.org) in April 2020, and encompass all mycobacteriophage clusters. The 115 known *Mycobacteriaceae* plasmids (Table S3) and 35 114 complete plasmids from non-* Mycobacteriaceae* families were obtained from the NCBI nucleotide database (https://www.ncbi.nlm.nih.gov/nuccore; searching for the term ‘complete plasmid’ using the filters: genomic DNA/RNA, RefSeq, Plasmid) in May 2020 and January 2020, respectively.

### Detection of putative mobilome elements

The identification of putative plasmids was performed by using four strategies. (i) blastn analyses of the contigs of the *Mycolicibacterium* genomes against 35 114 plasmid sequences obtained from the NCBI database. Those contigs presenting at least 50 % coverage and identity with NCBI plasmids were selected. (ii) Replication proteins were sought for in the proteomes of the *Mycolicibacterium* genomes using an in-house hmm profile with the hmmsearch program from the HMMER v3.1b2 software package [34]. This hmm profile was built using Rep proteins from *Mycobacteriaceae* plasmids with the hmmbuild program from the HMMER software [34], and it is available as Supplementary File 2. (iii) Circularity inference of the *Mycolicibacterium* contigs: assembly programs only produce linear contigs, so if a single contig has sufficient coverage with reads overlapping its ends, it is evidence of its circularity [35]. (iv) Searches for T7SSs related to plasmids because plasmid T7SS sequences differ from those encoded on chromosomes [13]. The T7SS core proteins (EccA, EccB, EccC, EccD, EccE and MycP) were surveyed by the hmmsearch program [34] using the hmm profiles listed in Table S4. To optimize the survey, we made a preliminary selection of the *Mycolicibacterium* contigs considering the MycP protein as a marker, as it is one of the most conserved proteins in the T7SS. The MycP sequences were clustered with MycP proteins from *Mycobacteriaceae* plasmids and chromosomes by the CD-HIT software v4.7 [36] using the parameter *-c 0.7*. Final selection of the *Mycolicibacterium* contigs that encoded plasmid T7SS was based on the clustering of their MycP with non-chromosomal MycP and the presence of at least four T7SS core genes close to each other. The hmmsearch and blastn searches of this study were performed using an e-value of 1e-10. All candidate contigs selected by the four strategies followed a filtering step to reduce the number of false-positives, discarding: (i) contigs with a sequence size less than 1 kb or greater than 1 Mb, (ii) contigs that encode ribosomal proteins and (iii) contigs that did not encode proteins. After these steps, the remaining contigs were considered potential plasmids.

The identification of putative prophages in the *Mycolicibacterium* genomes was performed using the PHASTER web platform (https://phaster.ca). The identified prophages were categorized as ‘intact’, ‘questionable’ and ‘incomplete’, depending on the number of phage-related encoded proteins and the prophage size [37].

### Mobilome annotation

The putative mobilome elements were annotated using Prokka v1.12 [38] with bacterial genetic code and *--rfam* parameters. The gene families present in the mobilome elements were defined by orthology analysis using GET_HOMOLOGUES v3.0.5 [39] with the parameters *-M -X -C 0.7 S 0.4 t 0*. Functional annotation of the predicted proteins was performed by InterProScan v5.42–78.0 [40] with default parameters. The survey of antibiotic resistance genes was based on The Comprehensive Antibiotic Resistance Database (https://card.mcmaster.ca) [41] and hmm core profile from the ResFams database [42]. Virulence factors were also surveyed with protein sequences of the core dataset of the Virulence Factor Database (VFDB) [43] using blastp with e-value, identity and coverage of 1e-10, 50 % and 70 %, respectively.

The genomic relatedness between the predicted plasmids and known *Mycobacteriaceae* plasmids (Table S3) was assayed by the combination of genome-wide average nucleotide identity (gANI) and alignment fraction (AF) using the MiSI (Microbial Species Identifier) method [44]. Plasmids were considered related if they presented pairwise AF ≥0.6 and pairwise gANI ≥96.5. Although this clustering method was originally applied for comparisons of bacterial species, it can also be used as a schematic measure to group genetically equivalent plasmids [45]. These data were embodied in heatmaps, created with the pheatmap R package (https://cran.r-project.org/web/packages/pheatmap).

The mobility prediction of the predicted plasmids was based on the presence of gene markers, such as relaxase and T4SS-like genes (*vir*B4 and T4CP). In addition, in *Mycobacteriaceae *, the ssDNA conjugative mechanism is also related to other genes, such as the T7SS and *tcp*C gene (*vir*B8-like conjugative gene) [46]. Thus, the predicted plasmids were classified as: (i) conjugative, plasmids encoding Relaxase, TcpC, VirB4, T4CP proteins, and the T7SS; (ii) mobilizable, plasmids encoding Relaxase, and lacking the T4SS and/or T7SS; and (iii) non-mobilizable, plasmids that did not encode Relaxase. We also checked whether the predicted plasmids could be in fact AICEs looking for replication-, integration- and conjugation-related genes as described [47]. The mobility markers were surveyed with hmm profiles (Table S4) using the hmmsearch program [34]. OriT sequences (origin sites of DNA transfer) were obtained from oriTDB [48], and screened on the predicted plasmids using blastn. The Relaxase sequences were also used for phylogenetic analysis, being aligned with MAFFT v7.310 [49] and submitted to PhyML v3.1 [50] for maximum-likelihood analysis with the WAG+I+G+F substitution model and 100 bootstrap replicates.

The clustering of prophage sequences was done with VSEARCH v2.14.2 [51] considering an identity ≥70 % and coverage ≥50 % (parameters *--strand both*, *--id 0.7*, *--query_cov 0.5* and *--target_cov 0.5*). Assignment of the prophages to a mycobacteriophage cluster was based on the similarity of its gene content with other mycobacteriophages (≥50 %) [52]. Thus, a bipartite network of the gene content of the prophages was generated by AcCNET v1.216 [53] using the parameters *--threshold 1.1* and *--kp ′-s 1.80 -e 1e-10 -c 0.6*′, and visualized in Cytoscape v3.7.2 [54].

### *Mycolicibacterium* and T7SS phylogeny

*Mycolicibacterium* and T7SS phylogeny were determined based on *Mycolicibacterium* core genes and T7SS proteins, respectively. The genus phylogeny was based on the core genome multilocus sequence analysis (cgMLSA) of the 242 *Mycolicibacterium* genomes using GET_HOMOLOGUES v3.0.5 [39] with the parameters *-M -X -C 0.7 S 0.4 t 0*. Plasmids encoding at least four of the six T7SS proteins, whose genes were close to each other, were selected for the T7SS phylogeny. The *Mycolicibacterium* core genes and the proteins encoded by the T7SS core genes in the predicted plasmids were aligned with MAFFT v7.310 [49] and low-quality alignment columns were removed by GUIDANCE2 v2.02 [55]. The final alignments of each data set (* Mycolicibacterium* genes and T7SS proteins) were concatenated and submitted to PhyML v3.1 [50] for maximum-likelihood analysis with GTR+G+I and LG+I+G+F substitution models, respectively, and 100 bootstrap replicates. The trees were generated in the iTOL web platform (https://itol.embl.de) [56].

### Statistical analysis

Statistical analysis between groups was performed with Wilcoxon tests using RStudio software v1.2.5033 [57], and *P* values <0.05 were considered statistically significant.

## Results and Discussion

This study focused on the mining of metagenomes (set of contigs), represented by complete and draft sequences from the genus *Mycolicibacterium *, to access their mobilome (plasmids and prophages). This strategy allowed the identification of a plethora of putative mobile elements, mainly plasmids, as well as a dozen intact prophages, significantly expanding the previous scenario of occurrence of these elements within this genus of bacteria.

### Plasmid detection and distribution among *Mycolicibacterium*

**Fig. 1.**
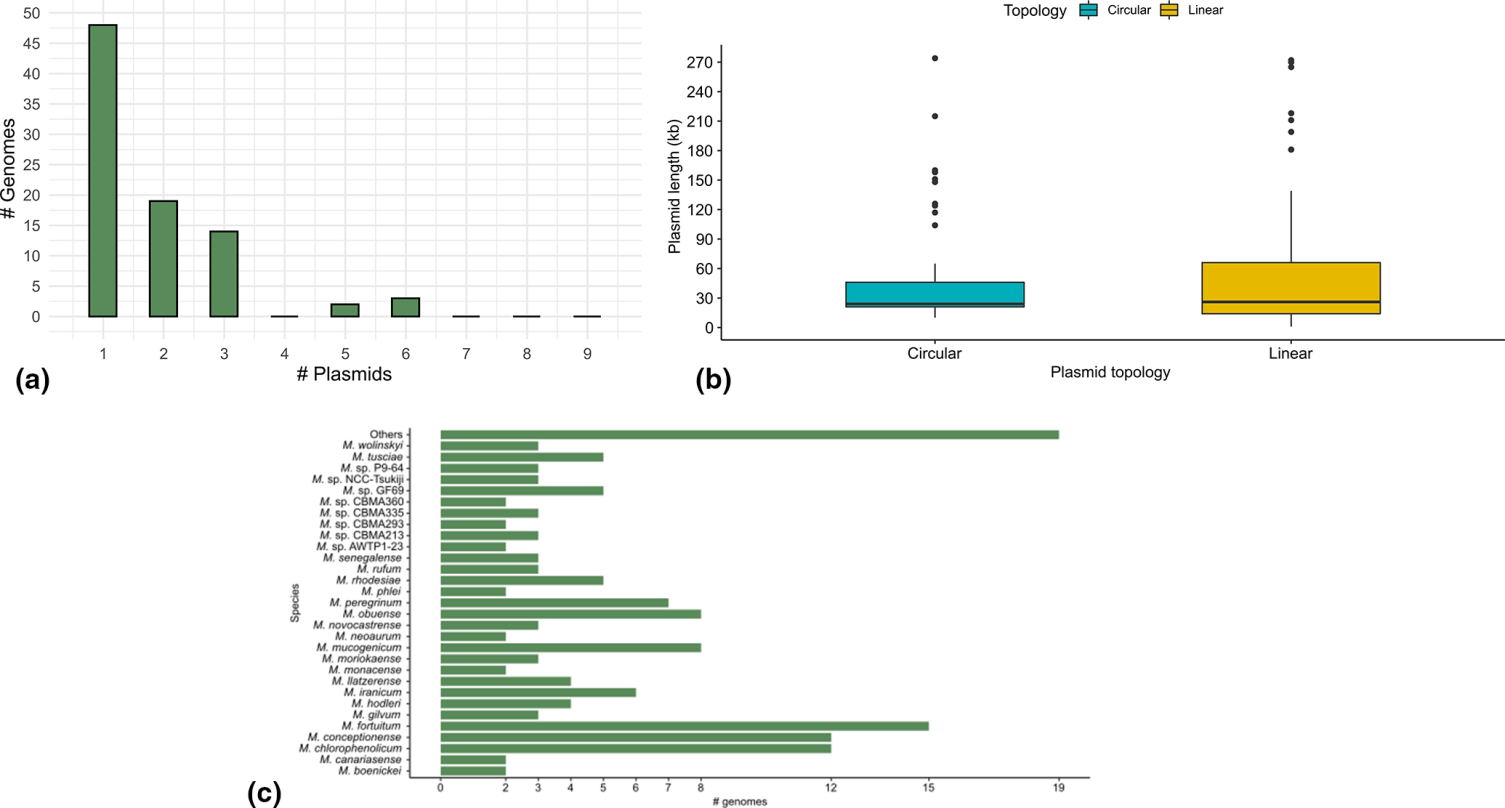
Characterization of *Mycolicibacterium* mobile elements. (**a**) Number of plasmids distributed in the genomes; (**b**) size distribution of the predicted plasmid according to their topology; (**c**) number of predicted plasmids identified in *Mycolicibacterium* species.

In *Mycobacteriaceae *, plasmids have been previously considered to be rare, most of them identified in clinical strains from the genus *Mycobacterium* [9, 10]. We therefore tested the hypothesis that the occurrence of plasmids in the family *Mycobacteriaceae* has been underestimated. We applied four *in silico* strategies versus 242 metagenomes (set of contigs) from 69 species and 27 sp. genomes of the genus *Mycolicibacterium *. In this way, we were able to identify 156 putative plasmids (Table S5) within 86 genomes (~35 % of 242). Most of these genomes presented a single plasmid (*n*=48), while the others contained two to six plasmids per genome (Fig. 1a). The sequence length of the predicted plasmids ranged from 1375 to 274 109 bp with a mean and median of 53.9 and 24.6 kb, respectively; they presented high GC content (66 % median), and 53/156 of them were predicted to have a circular topology (Fig. 1b). These features are similar to those observed in known *Mycolicibacterium* plasmids: sequence length ranging from 1474 to 615 278 bp, with a median of 214 kb (presence of megaplasmids is common); high GC content (65.15 % median); the presence of both circular and linear topologies, with a prevalence of the latter; and multiple plasmids (one to three) per genome. The high GC content is also similar to the chromosomal GC content of *Mycolicibacterium *. The discovery of this large set of putative plasmids in the current data set contrasts with a previous study that identified a low abundance of mobile elements in genomes of representative species of *Mycobacteriaceae* species, including some *Mycolicibacterium* [58].

**Fig. 2.**
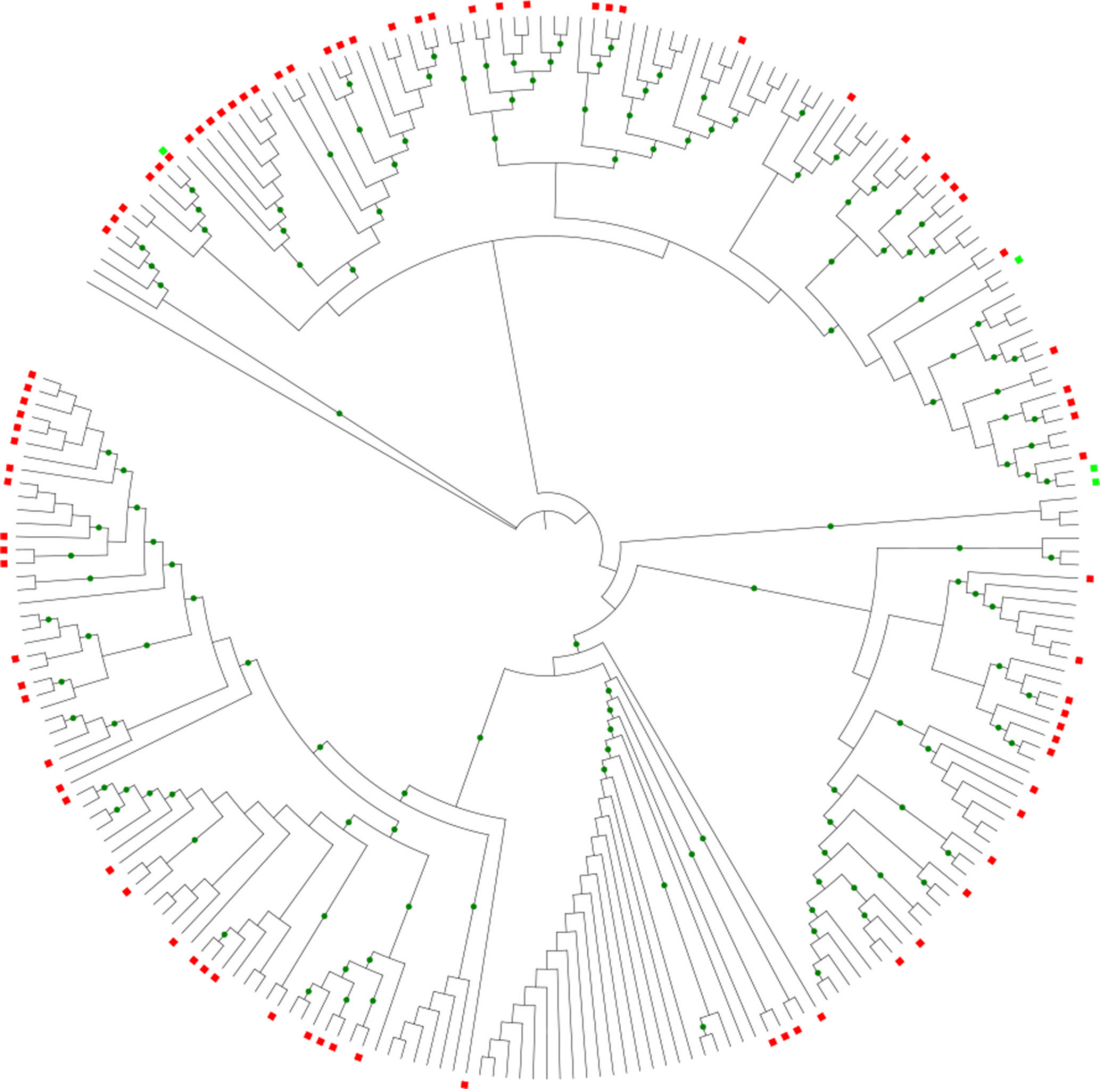
*
Mycolicibacterium* phylogenetic tree based on seven concatenated core genes of the genus (~8 kb): RNA polymerase sigma factor (*hrd*B-like), adenylate kinase (*adk*-like), 50S ribosomal subunit L19 (*rpl*S-like), glycogen accumulation regulator (*gar*A-like), riboflavin synthase (*rib*E-like), glutamine-hydrolysing carbamoyl-phosphate synthase small subunit (*car*A-like), and UMP kinase (*pyr*H-like). Genomes presenting the predicted plasmids and the known plasmids are marked with red and green squares, respectively. Green circles at branches indicate bootstrap values >80 %.

Among all genomes from the 69 *Mycolicibacterium* species, we observed that the plasmids were distributed in 36 species and 13 sp. genomes (>50 % of the total species), with a prevalence in *Mycolicibacterium fortuitum *, *Mycolicibacterium chlorophenolicum* and *Mycolicibacterium conceptionense* (Fig. 1c). Some species were represented by several genomes, and in these cases, plasmids were not always detected in all genomes from the species (Table S6). It is thus not possible to state that plasmids are common in these species. In the NCBI database, there are 11/86 *Mycolicibacterium* species harbouring plasmids, and now more 23 species have also been revealed carrying plasmids (Table S5). In fact, a phylogenetic analysis based on seven concatenated core genes (*hrd*B-like, *adk*-like, *rpl*S-like, *gar*A-like, *rib*E-like, *car*A-like and *pyr*H-like) of the genus (obtained by cgMLSA of the 242 *Mycolicibacterium* genomes) showed that there is a large distribution of plasmids among several species of the genus (Fig. 2). These results show that plasmids are prevalent in the newly defined genus *Mycolicibacterium *. Interestingly, most of these saprophyte species exploit environmental niches [1], in contrast to the previous scenario in which *Mycobacteriaceae* plasmids were characterized mainly in pathogenic strains [59, 60].

### *Mycolicibacterium* plasmid clustering and characterization

**Fig. 3.**
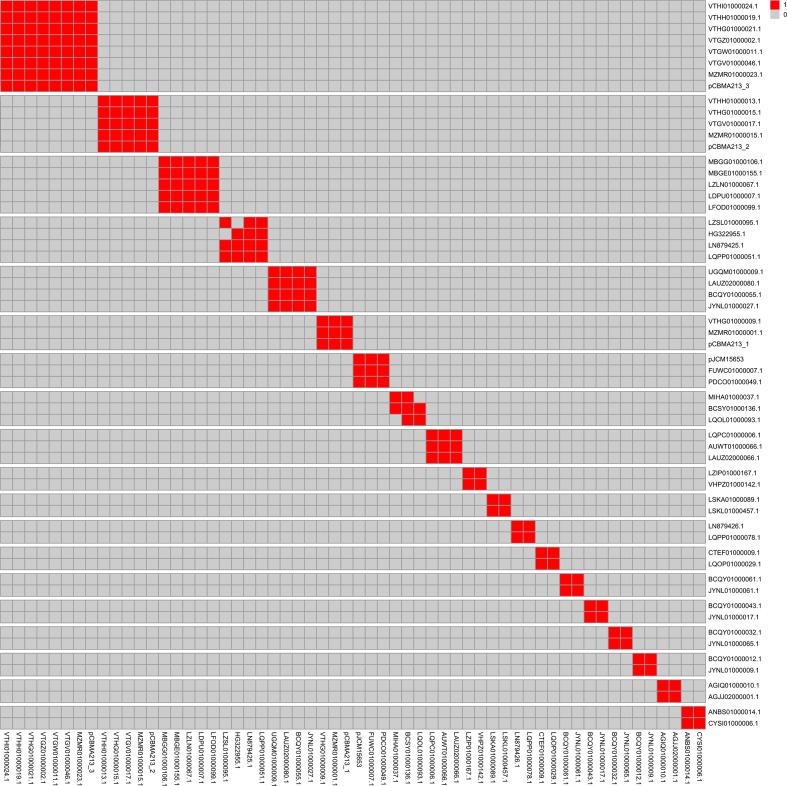
Heatmap showing groups of related plasmids. The pair of plasmids with a value of 1 (red) simultaneously present gANI≥96.5 and AF≥0.6. This analysis included all known *Mycobacteriaceae* plasmids (Table S3), but only those related to the predicted plasmids are displayed.

To observe the genomic relationship and the diversity among *Mycolicibacterium* plasmids, we compared the sequences of the predicted plasmids with the sequences of a representative set of known *Mycobacteriaceae* plasmids based on pairwise gANI and AF. Most of the predicted plasmids had no or only a low similarity to known *Mycobacteriaceae* plasmids (Figs S1 and S2). Fifty-four predicted plasmids formed 19 clusters of two to seven sequences (Fig. 3), of which 4/19 clusters showed an overall similarity to known *Mycolicibacterium* plasmids (pCBMA213_1, pCBMA213_2, pCBMA213_3 and pJCM15653). Although the overall genomic relationship of the predicted plasmids is limited to only these four known *Mycobacteriaceae* plasmids, when considering the *rep* gene, it was possible to observe phylogenetic relationships with several other known plasmids (Fig. S3). This shows some degree of distribution of plasmid replicon systems within the family *Mycobacteriaceae* as similar *rep* genes are present in plasmids of different species and genera. Thus, *Mycolicibacterium* plasmids with promiscuous replicon systems could be evaluated as potential cloning vectors within the family, as they come from non-pathogenic and fast-growing organisms (suitable for use in biotechnology). In addition, they could have applications in mycobacterial genetic manipulation, as they are not derived from hybrid vectors (*Mycobacterium–Escherichia coli*) [61, 62]

**Fig. 4.**
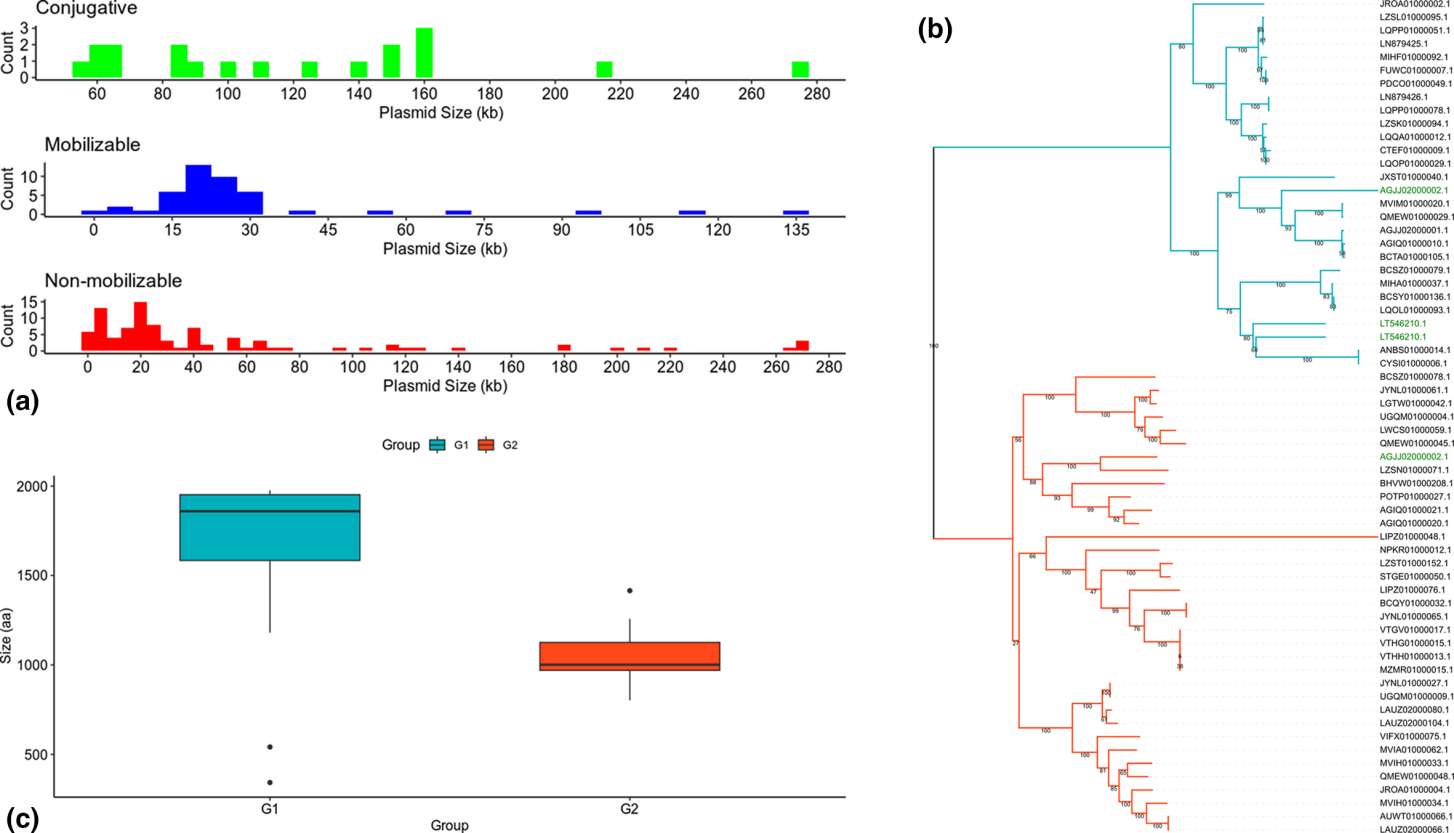
Mobility features of the predicted plasmids. (**a**) Number of plasmids versus their sequence size (kb) according to plasmid mobility. (**b**) Phylogeny of MOB_F_ relaxases encoded by the predicted plasmids. The blue and red branches represent the G1 and G2 relaxase groups, respectively. The green labels represent the plasmids that encode two relaxase genes. (**c**) Size of the relaxase proteins encoded by the predicted plasmids.

Within the 19 clusters, it was observed that plasmids from the same speciesand from a mix of species may or may not have a different geographical origin (Table 1). These results are evidence of the nature and mobility of these elements. The mobility of these plasmids was predicted based on gene marks (see Methods) and, in this way, 19/156 plasmids would be conjugative, 45/156 mobilizable and 92/156 non-mobilizable (Fig. 4a). The classification of plasmids as conjugative was based on the presence of the following set of genes: relaxase, *vir*B4, *vir*D4 (T4CP), *tcp*C and T7SS, as these genes were shown to be experimentally necessary for conjugation in *Mycobacteriaceae* [46]. This was a very strict classification and, therefore, we do not rule out the possibility of false negatives, as there may be conjugative systems not yet characterized in the family *Mycobacteriaceae *. Conjugative plasmids had a median sequence size of ~111 kb (thus representing megaplasmids), being significantly larger than mobilizable and non-mobilizable plasmids (*P*<0.05), which did not show significant differences in median size (~22 and ~23 kb, respectively). In fact, conjugative plasmids have already been observed to be larger than mobilizable and non-mobilizable plasmids [63]. Interestingly, we were able to identify genes encoding FtsK-like DNA translocase (TraB-like) in four predicted plasmids (AGJJ02000003.1, QMEW01000047.1, QQBJ01000022.1 and RXJU01000029.1). This protein was involved with the transfer of dsDNA in a unique process in *Streptomyces* plasmids [64]. Therefore, we assigned these elements as conjugative, but this mechanism had not yet been described in *Mycobacteriaceae *, and experiments are needed to confirm that this mechanism also occurs in *Mycolicibacterium *.

**Table 1.** Groups of related plasmids

Cluster	Plasmid	Size (bp)	Species	Geographical location	Isolation source	Mobility
Cluster 1	VTHI01000024.1	21 646	* Mycolicibacterium* sp. CBMA 230	Brazil	Soil	Non-mobilizable
	VTHH01000019.1	21 648	* Mycolicibacterium* sp. CBMA 293	Brazil	Soil	Non-mobilizable
	VTHG01000021.1	21 848	* Mycolicibacterium* sp. CBMA 335	Brazil	Soil	Non-mobilizable
	VTGZ01000002.1	20 936	* Mycolicibacterium* sp. CBMA 361	Brazil	Soil	Non-mobilizable
	VTGW01000011.1	21 648	* Mycolicibacterium* sp. CBMA 311	Brazil	Soil	Non-mobilizable
	VTGV01000046.1	20 701	* Mycolicibacterium* sp. CBMA 360	Brazil	Soil	Non-mobilizable
	MZMR01000023.1	21 616	* Mycolicibacterium* sp. CBMA 213	Brazil	Soil	Non-mobilizable
Cluster 2	VTHH01000013.1	158,04	* Mycolicibacterium* sp. CBMA 293	Brazil	Soil	Conjugative
	VTHG01000015.1	158 04	* Mycolicibacterium* sp. CBMA 335	Brazil	Soil	Conjugative
	VTGV01000017.1	1512	* Mycolicibacterium* sp. CBMA 360	Brazil	Soil	Conjugative
	MZMR01000015.1	160 34	* Mycolicibacterium* sp. CBMA 213	Brazil	Soil	Conjugative
Cluster 3	MBGG01000106.1	3829	* Mycolicibacterium conceptionense* strain IS-2586	India	Human	Non-mobilizable
	MBGE01000155.1	3514	* Mycolicibacterium conceptionense* strain GA-1041	India	Human	Non-mobilizable
	LZLN01000067.1	3362	* Mycolicibacterium conceptionense* strain 1165613.5	Mozambique	Human	Non-mobilizable
	LDPU01000007.1	4509	* Mycolicibacterium senegalense* strain CK2	USA	Human	Non-mobilizable
	LFOD01000099.1	3316	* Mycolicibacterium conceptionense* strain MLE	USA	Human	Non-mobilizable
Cluster 4	LZSL01000095.1	13 990	* Mycolicibacterium setense* strain 852014–10208_SCH5295773	South Africa	–	Mobilizable
	HG322955.1	22 117	* Mycolicibacterium septicum* DSM 44393	–	–	Non-mobilizable
	LN879425.1	22 342	* Mycolicibacterium peregrinum* strain CSUR P2098	–	–	Mobilizable
	LQPP01000051.1	22 193	* Mycolicibacterium peregrinum* strain DSM 43271	Mexico	Human	Mobilizable
Cluster 5	UGQM01000009.1	23 908	* Mycolicibacterium gilvum* strain NCTC10742	UK	Human	Mobilizable
	LAUZ02000080.1	24 594	* Mycolicibacterium obuense* strain UC1	USA	Human	Mobilizable
	BCQY01000055.1	23 596	* Mycolicibacterium chlorophenolicum* JCM 7439	–	–	Non-mobilizable
	JYNL01000027.1	23 697	* Mycolicibacterium chlorophenolicum* strain DSM 43826	–	Soil	Mobilizable
Cluster 6	VTHG01000009.1	270,78	* Mycolicibacterium* sp. CBMA 335	Brazil	Soil	Non-mobilizable
	MZMR01000001.1	272 46	* Mycolicibacterium* sp. CBMA 213	Brazil	Soil	Non-mobilizable
Cluster 7	FUWC01000007.1	22 112	* Mycolicibacterium boenickei* strain CIP107829	–	–	Mobilizable
	PDCO01000049.1	22 061	* Mycolicibacterium boenickei* strain CCUG47580	–	–	Mobilizable
Cluster 8	MIHA01000037.1	25 046	* Mycolicibacterium flavescens* strain M6	USA	Human	Mobilizable
	BCSY01000136.1	27 325	* Mycolicibacterium canariasense* JCM15298	Japan	Human	Mobilizable
	LQOL01000093.1	27 188	* Mycolicibacterium canariasense* strain CCUG 47953	Spain	Human	Mobilizable
Cluster 9	LQPC01000006.1	29 814	* Mycolicibacterium iranicum* strain DSM 45541	Iran	Human	Non-mobilizable
	AUWT01000066.1	29 735	* Mycolicibacterium iranicum* UM_TJL	Malaysia	Human	Mobilizable
	LAUZ02000066.1	29 871	* Mycolicibacterium obuense* strain UC1	USA	Human	Mobilizable
Cluster 10	LZIP01000167.1	7624	* Mycolicibacterium fortuitum* strain 852002–51564_SCH6189132-b	South Africa	–	Non-mobilizable
	VHPZ01000142.1	7991	* Mycolicibacterium fortuitum* strain MTB7	Morocco	Human	Non-mobilizable
Cluster 11	LSKA01000089.1	6012	* Mycolicibacterium mucogenicum* strain CCH10-A2	USA	hospital shower hose biofilm	Non-mobilizable
	LSKL01000457.1	4557	* Mycolicibacterium mucogenicum* strain CCH12-A2	USA	Hospital shower hose biofilm	Non-mobilizable
Cluster 12	LN879426.1	89 281	* Mycolicibacterium peregrinum* CSUR P2098	–	–	Conjugative
	LQPP01000078.1	83 406	* Mycolicibacterium peregrinum* strain DSM 43271	Mexico	Human	Conjugative
Cluster 13	CTEF01000009.1	13 398	* Mycolicibacterium conceptionense* D16	–	–	Mobilizable
	LQOP01000029.1	1381	* Mycolicibacterium conceptionense* strain CCUG 50187	Reunion: Indian Ocean	Human	Conjugative
Cluster 14	BCQY01000061.1	15 964	* Mycolicibacterium chlorophenolicum* JCM 7439	–	–	Non-mobilizable
	JYNL01000061.1	21 187	* Mycolicibacterium chlorophenolicum* strain DSM 43826	–	Soil	Mobilizable
Cluster 15	BCQY01000043.1	39 323	* Mycolicibacterium chlorophenolicum* JCM 7439	–	–	Non-mobilizable
	JYNL01000017.1	39 706	* Mycolicibacterium chlorophenolicum* strain DSM 43826	–	Soil	Non-mobilizable
Cluster 16	BCQY01000032.1	60 312	* Mycolicibacterium chlorophenolicum* JCM 7439	–	–	Conjugative
	JYNL01000065.1	6186	* Mycolicibacterium chlorophenolicum* strain DSM 43826	–	Soil	Conjugative
Cluster 17	BCQY01000012.1	265 87	* Mycolicibacterium chlorophenolicum* JCM 7439	–	–	Non-mobilizable
	JYNL01000009.1	270 77	* Mycolicibacterium chlorophenolicum* strain DSM 43826	–	Soil	Non-mobilizable
Cluster 18	AGIQ01000010.1	124 24	* Mycolicibacterium rhodesiae* JS60	USA	Aquifer sediment	Conjugative
	AGJJ02000001.1	111 84	* Mycolicibacterium tusciae* JS617	Germany	Groundwater	Conjugative
Cluster 19	ANBS01000014.1	71 255	* Mycolicibacterium mucogenicum* DSM 44124	–	–	Mobilizable
	CYSI01000006.1	95 450	* Mycolicibacterium mucogenicum* CSUR P2099	–	–	Mobilizable

Plasmids with distinct mobility signatures were observed within the 19 clusters (Table 1). All plasmids from Clusters 1, 3, 6, 10, 11, 15 and 17 were classified as non-mobilizable. Curiously, Cluster 3 encompasses plasmid sequences from different species, which suggests mobility, although they all were assigned as non-mobilizable. The presence of related ‘non-mobilizable’ plasmids between different species may be due to a *trans* mobilization mechanism, in which the oriT sequence (origin site of DNA transfer) is recognized by chromosomally encoded relaxases and/or by other mobile elements to initiate DNA transfer [65–69]. Thus, we searched among the predicted plasmids, using blastn, for putative oriT sequences using as reference hundreds of known oriT sequences obtained from an oriT sequence database (oriTDB). Although known oriT sequences have not been detected in the predicted plasmids, the existence of oriT sequences that have not yet been characterized cannot be excluded, because the *Mycobacteriaceae* plasmids and their features are beginning to be revealed. Besides, some Clusters (4, 5, 9 and 14) presented both mobilizable and non-mobilizable plasmids (Table 1). In particular, in Cluster 4, plasmid HG322955.1 was assigned as non-mobilizable, while the three others were assigned as mobilizable. By analysing the HG322955.1 sequence content we identified a relaxase, which would characterize it as a mobile plasmid, but this relaxase sequence lacks the TrwC domain, necessary for the DNA cleavage process, and essential to plasmid mobilization. In Clusters 5, 9 and 14, some plasmids seem to have lost their relaxases, as their related plasmids are of different species and have the relaxase gene. Clusters 2, 12 and 18 were composed entirely of conjugative plasmids (Table 1), and Cluster 13 presented conjugative and mobilizable plasmids. In this last group, the mobilizable plasmid (CTEF01000009.1) presented most of the genes of the conjugative apparatus, except the *tcp*C (*vir*B8-like) gene, so it was not assigned as conjugative according to our criteria.

Mobilizable and conjugative plasmids carry relaxases classified in several MOB families. In *Actinobacteria *, two relaxase families have been identified thus far: MOB_F_ and MOB_Q_ [70]. Here, the conjugative and mobilizable plasmids (64/156) presented 67 relaxase genes (three plasmids had two relaxase genes) that belonged to four MOB families: MOB_F_ (*n*=63), MOB_P_ (*n*=1), MOB_V_ (*n*=2) and MOB_C_ (*n*=1) (Table S7). Therefore, the spectrum of MOB families in *Mycolicibacterium* has been enlarged with the identification of MOB_P_, MOB_C_ and MOB_V_ in the current set of predicted plasmids. The MOB_F_ relaxases were the most prevalent and distributed in plasmids of several genomes, while the other MOB families were present in only three plasmids, all from a single genome (* Mycolicibacterium* sp. P9-64: NPKO01000025.1, MOB_V_; NPKO01000027.1, MOB_P_ and MOB_C_; and NPKO01000028.1, MOB_V_). These plasmids have some similarity to plasmids from *Firmicutes* and *Proteobacteria *, suggesting their broad host range profile. Indeed, *Actinobacteria* may be susceptible to broad-host-range plasmids, even under environmental conditions [71]. Performing a phylogenetic analysis with the protein sequences encoded by MOB_F_ relaxases, we observed two main clades (Fig. 4b) characterized by the size of their sequences (Fig. 4c), where those in G1 (1859 aa of median size) are larger than those in G2 (1001 aa of median size) (*P*<0.05). We did not observe any correlation between the relaxase groups and the mobility categories of the predicted plasmids. Likewise, analysing these relaxase protein sequences with InterProScan, we also did not observe any remarkable difference between the protein domains of the groups. The TrwC and P-loop_NTPase (DNA helicase-related) protein domains were the main ones identified in both relaxase groups, but some sequences from both groups also had variations, presenting one AAA+ATPase or two AAA_30 domains (Table S8). Interestingly, the AAA+ATPase domain is present in key proteins of single- and double-stranded DNA conjugation systems [72]. The presence of these additional domains suggests that some relaxases may play a role in various processes of mobilization and conjugation, or other unexpected roles [73].

Comparing the predicted plasmids (*n*=156) against 35 114 complete plasmids from non-* Mycobacteriaceae* families using blastn, it was revealed that 55/156 shared sequences (1–15 kb) with 139 plasmids of 34 genera from four phyla, including *Actinobacteria* (mainly), *Proteobacteria *, *Cyanobacteria* and *Firmicutes* (Table S9). It is evidence that plasmids play a role in the gene flux between *Mycolicibacterium* and other bacterial families. Indeed, horizontal gene transfer events between *Mycobacteriaceae* and *Proteobacteria* are common [45, 58, 74], but the association of these events with plasmids is unclear.

These results show that most of the predicted plasmids would represent new plasmids and therefore there is an as yet unravelled plasmid diversity in the genus *Mycolicibacterium *. Besides, the occurrence of related plasmids within several species from different geographical regions suggests their broad host range spectrum and the possibility of horizontal gene transfer among, at least, *Mycolicibacterium* species.

Interestingly, one of the predicted plasmids (BCTA01000038.1) could be an AICE, a class of integrative and conjugative elements (ICEs) prevalent in *Actinobacteria* [75], as it encodes an integrase, FtsK-like DNA translocase, and replicative genes. So far, there are few reports of AICEs in *Mycobacteriaceae* [47, 76]. In fact, due to the bias imposed by our approach that considered only contigs shorter than 1 Mb in size, the identification of AICEs in the integrated state would be unlikely. The identified element would be in the excised transient form, and the occurrence of these elements in *Mycolicibacterium* could still be underestimated. Plasmids and ICEs have similar genetic modules, such as replication, maintenance, segregation, etc., and, additionally, recombination and interconversion events between these elements seem to have been frequent during their evolution [77–81].

### Gene content of the putative plasmids

**Fig. 5.**
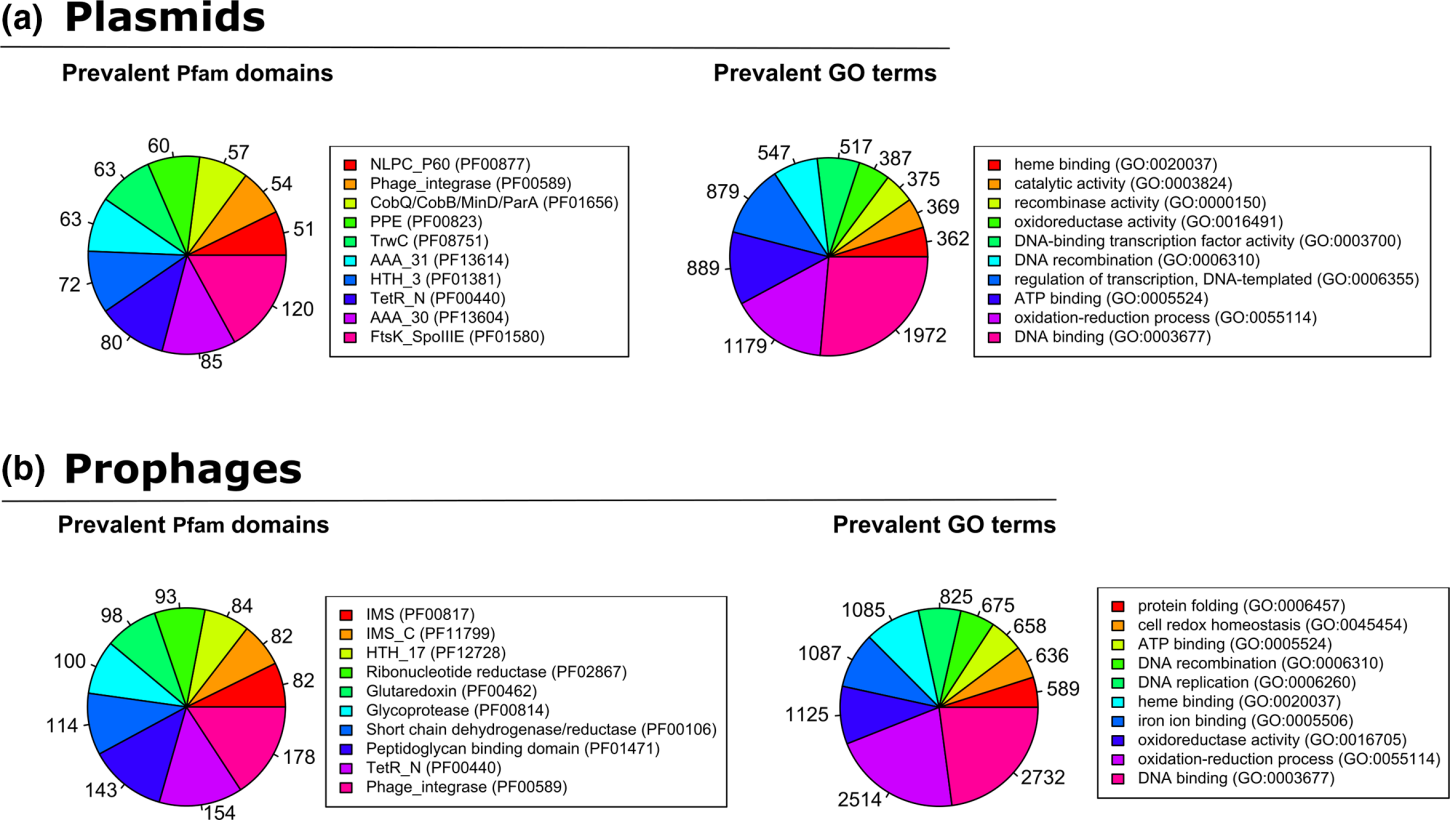
Functional characterization of the proteins encoded by the mobile elements. The number of proteins of the predicted plasmids (**a**) and prophages (**b**) presenting the most prevalent PF domains and GO terms.

**Fig. 6.**
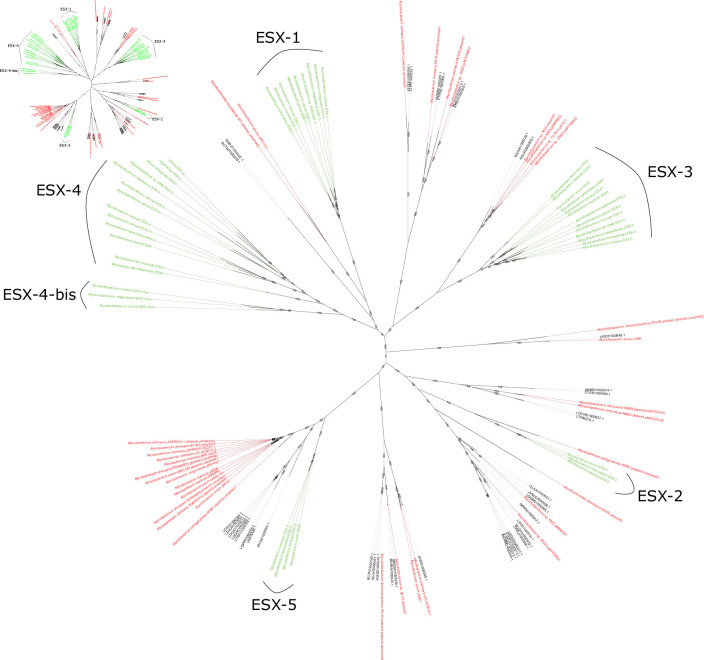
Maximum-likelihood tree of ESX loci encoded by *Mycobacteriaceae* plasmids. The reference sequences of chromosomal and plasmid origin are indicated and coloured in green and red, respectively.

To identify the diversity of genes carried by the predicted plasmids, we performed an orthology analysis, identifying 4643 gene families, most of them encoding uncharacterized proteins (in the same way as in known *Mycolicibacterium* plasmids). In general, the most prevalent gene families were those coding for replication (present in 71 plasmids), recombinase (*n*=42), toxin–antitoxin (TA) modules (*n*=39), relaxase (*n*=34), VirD4 (*n*=25) and MycP (*n*=25) proteins. Most of these genes are related to basal functions (replication, mobility and maintenance). Besides, by performing a functional analysis of the proteome of these plasmids (9137 proteins), around 25 % (*n*=2215) and 43 % (*n*=3884) of the proteins were assigned to Gene Ontology (GO) terms and Pfam protein domain composition, respectively (Table S10). The most prevalent Pfam domains and GO terms were mainly related to mobility, partitioning and translocation of macromolecules; and molecular functions, respectively (Fig. 5a). In addition to the cargo genes, some predicted plasmids (*n*=7) encoded tRNA genes (one to 32 tRNA genes). Although uncommon, some known plasmids also harbour tRNA genes [82–84], which can act as a target for integrative elements in recombination events [85]. We also observed in 40/156 (~25 %) of the predicted plasmids clusters of genes that resemble the T7SS. The T7SS is a common feature in *Mycobacteriaceae* plasmids, and in fact, they played a major role in the evolution and radiation of this secretion system in this bacterial family [11–13]. Among the predicted plasmids, those harbouring the T7SS had a median size (~117 kb) larger than plasmids that did not encode the T7SS (~21 kb) (*P* <0.05), and all ESX types were represented, except ESX-4, with most sequences encoding ESX-2 and ESX-5 (Fig. 6). The *Mycolicibacterium* and *Mycobacterium* ESX-5 sequences belong to two distinct clades, while there is no segregation of ESX-3 sequences related to these genera (Fig. 6). The plasmids harbouring ESX-1 and ESX-3 were predicted as non-mobilizable, while those harbouring ESX-2 and ESX-5 could be assigned as non-mobilizable, mobilizable or conjugative. In addition, an association of T4SS-like genes with the T7SS has been observed, mainly in plasmids harbouring ESX-2 and ESX-5, as most of them carried *vir*B4-like, *vir*D4-like and *tcp*C-like genes close to the T7SS loci, resembling a conjugation-related locus [13]. None of the putative plasmid sequences branched into chromosomal ESX clades, grouping mainly with sequences of known plasmids from different species of *Mycobacteriaceae *, which gives confidence that these sequences belong to mobile elements and reinforces the hypothesis of T7SS mobility [13]. Regarding the ESX types most commonly identified here, ESX-2 and ESX-5, they appear to be exclusive to plasmids, as there are no reports of them on *Mycolicibacterium* chromosomes [11–13]. While *Mycolicibacterium* plasmids carrying ESX-2 are common, those with ESX-5 have rarely been observed [13]. Therefore, our analysis revealed a new scenario considering the prevalence and distribution of this ESX system in *Mycolicibacterium *. Curiously, ESX-1 and ESX-3 were only identified in non-mobilizable plasmids, while ESX-2 and ESX-5 were found in plasmids of all types of mobility. We speculate that the presence/absence of the different ESX types in *Mycolicibacterium* plasmids could have a relationship to their role and evolutionary time. ESX-4 is the most ancestral, being already chromosomally fixed in the species, and is involved in lateral gene transfer [13], not being observed in *Mycolicibacterium* plasmids. ESX-1 and ESX-3 arose after ESX-4, and have been identified particularly in the chromosome, being involved in lateral gene transfer and iron acquisition, respectively [13]. So far, only a few *Mycolicibacterium* plasmids are known to harbour these ESX systems. Conversely, ESX-2 and ESX-5, the most phylogenetically recent ESX systems, are only found in plasmids. ESX-5 is related to virulence and membrane integrity processes, while the function of ESX-2 is unknown [13]. Thus, more ancestral ESX systems would have been fixed by a selection of their functions, no longer diversifying as much in the plasmids (observed by the lower number of plasmids carrying ESX-1 and ESX-3), while more recent ESX systems (ESX-2 and ESX-5) would still be under selection and have not yet been fixed.

In bacteria, plasmids are one of the main vectors for the spread of antibiotic resistance genes, and represent an example of bacterial adaptation in response to selective pressures [86–90]. Among the identified genes from the accessory genome of the 156 predicted plasmids, 184 genes were related to antibiotic resistance of 27 drug classes. Most of these genes were classified as ‘Loose’ [91], presenting low identity (20–46 %) to the reference sequences of the CARD database (Table S11), which suggests a distant homology. However, two sequences (LSKL01000015.1_00012 and LSKL01000015.1_00027), from a single non-mobilized plasmid of *Mycolicibacterium mucogenicum *, encoded proteins with higher identity (76 %) to proteins associated with aminoglycoside resistance. Most of the antibiotic resistance mechanisms predicted were associated with antibiotic efflux and antibiotic target alteration (Table S11). As these sequences could be distant homologues of clinical reference sequences, we further analysed them, focusing on those with an enzymatic modification mechanism (antibiotic inactivation), looking for functional domains using hmm profiles from the ResFams database. Among the 184 antibiotic resistance genes identified by CARD, 12 (~6 %) were associated with antibiotic inactivation, but only three presented functional domains listed in the ResFams database (Table S11, genes marked in red). All these genes were distributed in 58 plasmids harboured by 46 *Mycolicibacterium* strains, of which 31 had data on their sources, and most of them (*n*=19) were isolated from the environment (mainly soil and water), and the rest (*n*=12) from clinical samples. The distant homology of the genes related to antibiotic resistance harboured by *Mycolicibacterium* plasmids suggests their role as a reservoir of resistance genes that could later emerge in the clinic [91–93]. In addition to antibiotic resistance genes, some plasmids (e.g. LDPU01000005.1, ANBS01000014.1 and CYSI01000006.1) harboured whole operons related to mercury and arsenic resistance, and blastn analyses showed their presence both on chromosomes and on plasmids from other species and genera of bacteria (in some cases with high identity: ~90 %).

We also looked for virulence factors in the gene content of the predicted plasmids through the VFDB database. Although we did not identify any type of toxin in the predicted plasmids, most of the results indicated some T7SS genes (e.g. *myc*P, *ecc*A, *esx*A, *esx*G, *esx*H) as virulence factors. Indeed, the T7SS has already been associated with pathogenicity in the genus *Mycobacterium *, mainly by the secreted proteins EsxA, EsxB, PE and PPE [94]. However, this was only observed in mycobacterial pathogens (e.g. *Mycobacterium marinum *, *Mycobacterium bovis *, *Mycobacterium tuberculosis *), as in *Mycolicibacterium smegmatis* the same T7SSs did not induce the pathogenic profile [95, 96]. In fact, here, these secreted proteins (EsxA, EsxB, PE, PPE) encoded in the predicted plasmids showed low identity to the proteins encoded by mycobacterial pathogens (~20–40 %). However, the current knowledge of PE/PPE diversity suggests that members of this large and diverse family of proteins may be associated with the evolution of pathogenicity [97]. In addition, these studies focused on the T7SS of chromosomal origin, and thus, despite the presence of distinct T7SSs in *Mycolicibacterium* plasmids, their activities, concerning other functions than conjugation [46], are still unknown. The association of a plasmid with pathogenicity has already been reported in the family *Mycobacteriaceae* [98], but to date, there are no experimental studies with plasmids focusing on the pathogenesis of *Mycolicibacterium *. Therefore, because *Mycolicibacterium* is a saprophytic genus and, occasionally an opportunistic pathogen, the accessory genes of its plasmids are more likely to play a role in their adaptation and survival in the environment.

### Identification and characterization of *Mycolicibacterium* prophages

**Fig. 7.**
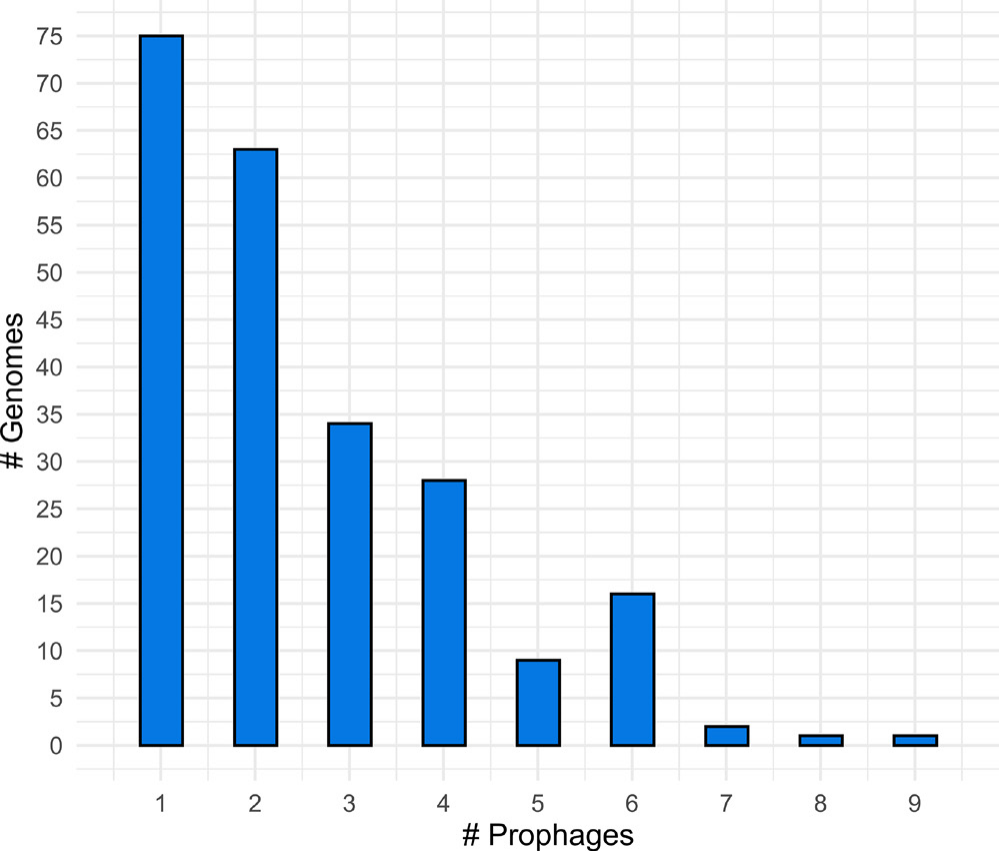
The number of prophages distributed in the genomes.

Mycobacteriophages are commonly identified in *Mycobacteriaceae *, with reports of more than 11 000 isolated phages and more than 1800 completely sequenced (http://phagesdb.org) using the model organism *M. smegmatis* mc^2^155 [31–33]. Despite the enormous number of phages identified, there is a lack of studies on their occurrence in other *Mycolicibacterium* species [99–101]. Using PHASTER, a total of 566 prophages were predicted in 229/242 *Mycolicibacterium* genomes (~94 %) (File S4). Their sequence size ranged from 3406 to 112 372 bp with a mean and median of ~16.4 and 10 kb, respectively. Similar to the plasmids, most genomes had a single element, while the others encoded two to nine prophages per genome (Fig. 7). Among the 566 prophages, only 40 were assigned as intact prophages, 34 as questionable, and the rest as cryptic prophages (incomplete). The 566 prophages were distributed in 66/69 *Mycolicibacterium* species, besides 26/27 sp. genomes. Genomes from *Mycolicibacterium chitae *, *Mycolicibacterium phocaicum* and *Mycolicibacterium pyrenivorans* did not present prophages; however, this could be a result of sample bias, as each of these species has only one genome available. Performing a clustering analysis, the 566 prophages formed 345 clusters, which consisted mainly of prophages of bacteria of the same species. The intact prophages only clustered with other intact and/or questionable prophages (Table S12). Therefore, the evidence raised here suggests that mycobacteriophages are abundant mobile elements in most of the identified species of the genus (~94 % of the genomes presented prophages). These prophages showed great diversity (considering the number of clusters), but most of them were assigned as cryptic. In this case, they may represent archaeological remnants that have been domesticated by their hosts [102].

### Gene content of the prophages

Gene content analysis of the 566 prophages revealed a pan-genome composed of 5177 genes, of which the most prevalent were those encoding for redoxin NrdH (present in 90 prophages), DNA polymerase IV (*n*=81), class Ib ribonucleoside-diphosphate reductase assembly flavoprotein NrdI (*n*=81), class 1b ribonucleoside-diphosphate reductase subunit alpha (*n*=80) and SDR family oxidoreductase (*n*=79). Functional analysis of their proteome (*n*=12 457 proteins) could only assign ~27 % (*n*=3390) and ~40 % (*n*=5055) to GO and Pfam terms, respectively (Table S13). The most prevalent proteins were associated with biological and molecular processes related to phage protection, integration and survival (Fig. 5b). Among the genes related to survival, the impB/mucB/samB family (IMS) is related to UV protection [103], which is the major cause of phage mortality in marine environments, at least near the surface [104]. In addition, several proteins have been associated with haem and iron-binding processes, which can favour the lytic process [105]. Besides protein-coding genes, 61 prophages encoded one (*n*=43), two (*n*=7), three (*n*=4) or four (*n*=7) tRNA genes; and 42 prophages encoded other non-coding RNA (ncRNA), including: ALIL (*n*=18), SAM-IV (*n*=16), TPP (*n*=4), tmRNA (*n*=3), Ms_AS-8 (*n*=2), Intron_gpII (*n*=1), ncRv12659 (*n*=1) and YrlA (*n*=1). This great diversity of ncRNA genes related to regulatory processes [106–109] may reflect the amplitude of the metabolic processes of these prophages and, in fact, tRNA genes are a common feature in mycobacteriophages, their presence being explained based on the codon/amino acid usage [110, 111].

In the survey of antibiotic resistance genes, 241 genes related to 29 classes of drugs were identified in 175 prophages, most of them presenting low identity (22–64 %) to the reference sequences of the CARD database (Table S14). As with the plasmids, we assessed the functional domains in the antibiotic inactivation genes identified in the prophages. Among the antibiotic resistance genes analysed, 73 (~30 %) were associated with antibiotic inactivation (most of them related to beta-lactams and aminoglycosides), of which 22 presented functional domains listed in the ResFams database (Table S14, genes marked in red). Thus, they can also play a role as a reservoir, with a further impact on their host’s fitness [93, 112, 113]. The exception was the cryptic *Mycolicibacterium goodii* X7B prophage encoding an *arr* gene with high similarity (92 % identity) to a functional gene of *M. smegmatis *. In fact, the *arr* gene was first identified on the chromosome of one *M. smegmatis* strain [114], a species closely related to *M. goodii *, and more recently, it has been reported in several species of *Actinobacteria *, as well as in mobile elements of *Proteobacteria* [115]. However, currently, this prophage seems to be domesticated and degraded, as no structural gene was found next to the *arr* gene. This provides evidence for the role of mycobacteriophages in the dissemination of genes in this genus.

### Mycobacteriophage cluster designation

**Fig. 8.**
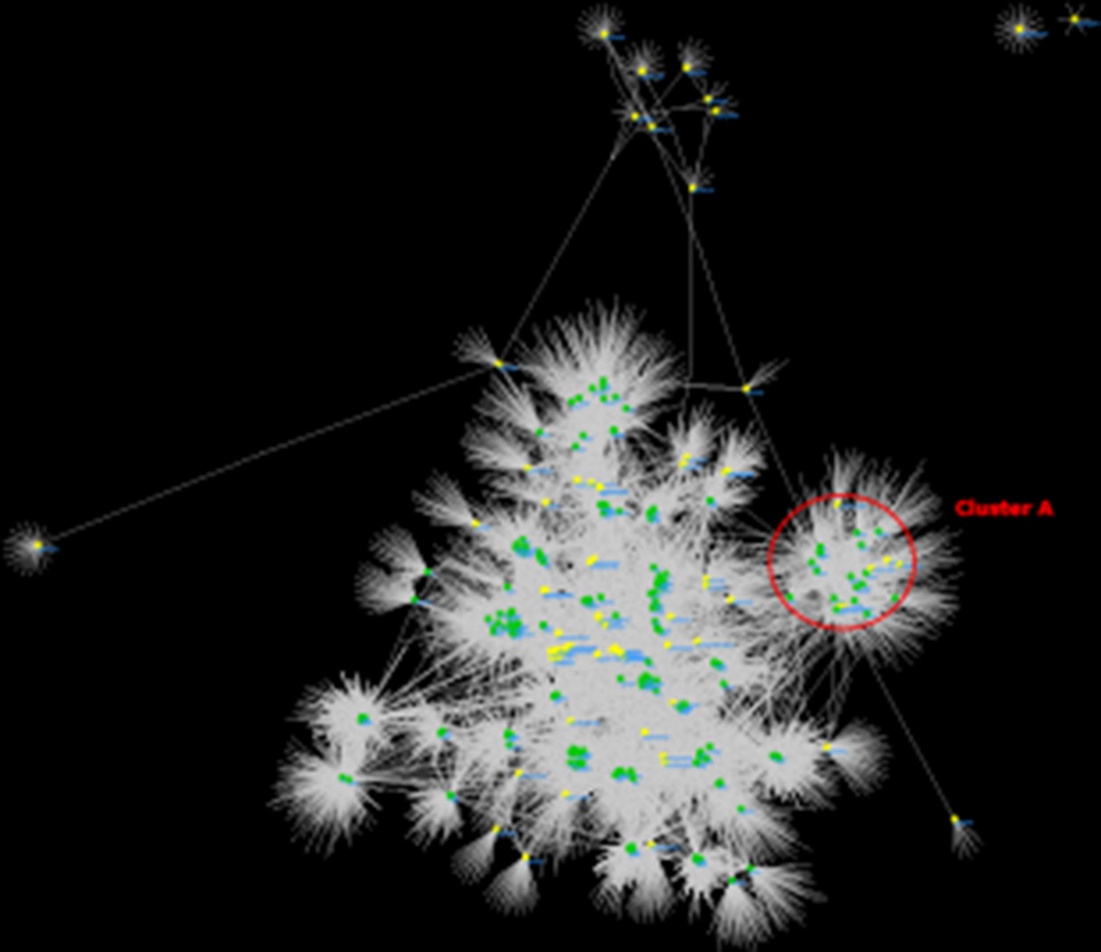
The bipartite network of the gene content of the prophages. Coloured circles and grey diamonds represent the genomes and the genes, respectively. Edges connect the genomes to the genes carried by them. The green and yellow circles represent the reference mycobacteriophages and the predicted prophages, respectively. Mycobacteriophages belonging to cluster A are delimited by the red circle.

We performed a bipartite network analysis using the gene content of the intact and questionable prophages (Table S15) to classify them into mycobacteriophage clusters. Most of these prophages (34/40) did not share at least 50 % of their gene content with the reference mycobacteriophages, and therefore they could not be assigned to a recognized cluster. On the other hand, 6/40 could be clustered with cluster A mycobacteriophages (Fig. 8). In addition, two *Mycolicibacterium senegalense* prophages clustered with a singleton phage (Sparky), which could represent a new mycobacteriophage cluster. Although the designation of a cluster is based on phages obtained experimentally and then sequenced [31], the clusters of prophages identified here may represent new clusters of mycobacteriophages.

## Conclusion

Through these extensive analyses, it was possible to test and confirm the hypothesis regarding the underestimation of plasmids and prophages in a genus of the family *Mycobacteriaceae *. Initially, 19 plasmids were assigned to *Mycolicibacterium* and now dozens of this mobile element are described in the genus, and similarly, prophages have been identified in several species. Also, evidence has been raised regarding the role of mobile genetic elements in the diversity and evolution of *Mycolicibacterium *, as they can carry genes associated with secretion systems, resistance to metals and antibiotics, and modulation of functions that affect survival and virulence, as PE/PPE gene families.

## Supplementary Data

Supplementary material 1

Supplementary material 2

## References

[1] Gupta RS, Lo B, Son J (2018). Phylogenomics and comparative genomic studies robustly support division of the genus *mycobacterium* into an emended genus *mycobacterium* and four novel genera [published correction appears in Front Microbiol. 2019 Apr 09;10:714]. Front Microbiol.

[2] Morgado SM, Paulo Vicente AC (2020). Genomics of Atlantic forest *Mycobacteriaceae* strains unravels a mobilome diversity with a novel integrative conjugative element and plasmids harbouring T7SS. Microb Genom.

[3] Harrison E, Brockhurst MA (2012). Plasmid-Mediated horizontal gene transfer is a coevolutionary process. Trends Microbiol.

[4] Lorenzo-Díaz F, Fernández-López C, Lurz R, Bravo A, Espinosa M (2017). Crosstalk between vertical and horizontal gene transfer: plasmid replication control by a conjugative relaxase. Nucleic Acids Res.

[5] Kothari A, Soneja D, Tang A, Carlson HK, Deutschbauer AM (2019). Native plasmid-encoded mercury resistance genes are functional and demonstrate natural transformation in environmental bacterial isolates. mSystems.

[6] Rodríguez-Beltrán J, Sørum V, Toll-Riera M, de la Vega C, Peña-Miller R (2020). Genetic dominance governs the evolution and spread of mobile genetic elements in bacteria. Proc Natl Acad Sci U S A.

[7] Carr VR, Shkoporov A, Hill C, Mullany P, Moyes DL (2020). Probing the mobilome: discoveries in the dynamic microbiome [published online ahead of print, 2020 May 11]. Trends Microbiol.

[8] Durrant MG, MM L, Siranosian BA, Montgomery SB, Bhatt AS (2020). A bioinformatic analysis of integrative mobile genetic elements highlights their role in bacterial adaptation. Cell Host Microbe.

[9] Shintani M, Sanchez ZK, Kimbara K (2015). Genomics of microbial plasmids: classification and identification based on replication and transfer systems and host taxonomy. Front Microbiol.

[10] Gray TA, Derbyshire KM (2018). Blending genomes: distributive conjugal transfer in mycobacteria, a sexier form of HGT. Mol Microbiol.

[11] Dumas E, Boritsch EC, Vandenbogaert M, de la Vega RCR, Thiberge J (2016). Mycobacterial pan-genome analysis suggests important role of plasmids in the radiation of type VII secretion systems. Genome Biol Evol..

[12] Newton-Foot M, Warren RM, Sampson SL, van Helden PD, Gey van Pittius NC (2016). The plasmid-mediated evolution of the mycobacterial Esx (type VII) secretion systems. BMC Evol Biol..

[13] Mortimer TD, Weber AM, Pepperell CS (2017). Evolutionary thrift: mycobacteria Repurpose plasmid diversity during adaptation of type VII secretion systems. Genome Biol Evol.

[14] Kallimanis A, Karabika E, Mavromatis K, Lapidus A, Labutti KM (2011). Complete genome sequence of Mycobacterium sp. strain (Spyr1) and reclassification to Mycobacterium gilvum Spyr1. Stand Genomic Sci.

[15] Greninger AL, Cunningham G, JM Y, Hsu ED, Chiu CY (2015). Draft genome sequence of *Mycobacterium elephantis* strain LiPA. Genome Announc..

[16] Levasseur A, Asmar S, Robert C, Drancourt M (2016). Draft genome sequence of *Mycobacterium houstonense* strain ATCC 49403T. Genome Announc..

[17] de Man TJ, Perry KA, Lawsin A, Coulliette AD, Jensen B (2016). Draft genome sequence of *Mycobacterium wolinskyi*, a Rapid-Growing species of nontuberculous mycobacteria. Genome Announc.

[18] Bouam A, Robert C, Croce O, Levasseur A, Drancourt M (2017). Draft genome sequence of *Mycobacterium boenickei* CIP 107829. Genome Announc.

[19] Bouam A, Levasseur A, Drancourt M (2018). Draft genome sequence of *Mycobacterium porcinum* CSURP1564. Genome Announc.

[20] Fukano H, Yoshida M, Shouji M, Hatta S, Maruyama D (2019). Draft genome sequence of *Mycolicibacterium* sp. strain NCC-Tsukiji, isolated from blood culture of a patient with malignant lymphoma. Microbiol Resour Announc.

[21] Zsilinszky I, Gyula P, Bihari Z, Fehér B, Szabó Z (2019). Draft genome sequence of *Mycolicibacterium* sp. strain CH28, a potential degrader of diisopropyl ether, isolated from pharmaceutical wastewater. Microbiol Resour Announc.

[22] Komatsu T, Ohya K, Sawai K, Odoi JO, Otsu K (2019). Draft genome sequences of *Mycolicibacterium peregrinum* isolated from a pig with lymphadenitis and from soil on the same Japanese pig farm. BMC Res Notes..

[23] Nouioui I, Sangal V, Cortés-Albayay C, Jando M, Igual JM (2019). *Mycolicibacterium stellerae* sp. nov., a rapidly growing scotochromogenic strain isolated from *Stellera chamaejasme*. Int J Syst Evol Microbiol.

[24] Ong JFM, Tan LT (2019). Draft genome sequence of Mycolicibacterium sp. strain 018/SC-01/001, isolated from the marine sponge Iotrochota sp. Microbiol Resour Announc.

[25] Sánchez M, Blesa A, Sacristán-Horcajada E, Berenguer J (2019). Complete genome sequence of *Mycolicibacterium hassiacum* DSM 44199. Microbiol Resour Announc..

[26] Vatlin AA, Shur KV, Danilenko VN, Maslov DA (2019). Draft genome sequences of 12 *Mycolicibacterium smegmatis* strains resistant to imidazo[1,2-b][1,2,4,5]Tetrazines. Microbiol Resour Announc.

[27] Rauzier J, Moniz-Pereira J, Gicquel-Sanzey B (1988). Complete nucleotide sequence of pAL5000, a plasmid from *Mycobacterium fortuitum*. Gene.

[28] Bachrach G, Colston MJ, Bercovier H, Bar-Nir D, Anderson C (2000). A new single-copy mycobacterial plasmid, pMF1, from *Mycobacterium fortuitum* which is compatible with the pAL5000 replicon. Microbiology.

[29] Morgado SM, Marín MA, Freitas FS, Fonseca EL, Vicente ACP (2017). Complete plasmid sequence carrying type IV-like and type VII secretion systems from an atypical mycobacteria strain. Mem Inst Oswaldo Cruz.

[30] Ren L, Fan S, Wang J, Ruth N, Qiao C (2017). Complete genome sequence of a phthalic acid esters degrading *Mycobacterium* sp. YC-RL4. Braz J Microbiol.

[31] Hatfull GF (2018). Mycobacteriophages. Microbiol Spectr..

[32] Pope WH, Bowman CA, Russell DA, Jacobs-Sera D, Asai DJ (2015). Whole genome comparison of a large collection of mycobacteriophages reveals a continuum of phage genetic diversity. Elife.

[33] Singh S, Godavarthi S, Kumar A, Sen R (2019). A mycobacteriophage genomics approach to identify novel mycobacteriophage proteins with mycobactericidal properties. Microbiology.

[34] Eddy SR (2011). Accelerated profile HMM searches. PLOS Comp. Biol.

[35] Jørgensen TS, Xu Z, Hansen MA, Sørensen SJ, Hansen LH (2014). Hundreds of circular novel plasmids and DNA elements identified in a rat cecum metamobilome. PloS one.

[36] Fu L, Niu B, Zhu Z, Wu S, Li W (2012). CD-HIT: accelerated for clustering the next-generation sequencing data. Bioinformatics.

[37] Arndt D, Grant JR, Marcu A, Sajed T, Pon A (2016). PHASTER: a better, faster version of the PHAST phage search tool. Nucleic Acids Res.

[38] Seemann T (2014). Prokka: rapid prokaryotic genome annotation. Bioinformatics.

[39] Contreras-Moreira B, Vinuesa P (2013). GET_HOMOLOGUES, a versatile software package for scalable and robust microbial pangenome analysis. Appl Environ Microbiol.

[40] Jones P, Binns D, Chang HY, Fraser M, Li W (2014). InterProScan 5: genome-scale protein function classification. Bioinformatics.

[41] McArthur AG, Waglechner N, Nizam F, Yan A, Azad MA (2013). The comprehensive antibiotic resistance database. Antimicrob Agents Chemother.

[42] Gibson MK, Forsberg KJ, Dantas G (2015). Improved annotation of antibiotic resistance determinants reveals microbial resistomes cluster by ecology. Isme J.

[43] Chen L, Yang J, Yu J, Yao Z, Sun L (2005). VFDB: a reference database for bacterial virulence factors. Nucleic Acids Res.

[44] Varghese NJ, Mukherjee S, Ivanova N, Konstantinidis KT, Mavrommatis K (2015). Microbial species delineation using whole genome sequences. Nucleic Acids Res.

[45] Redondo-Salvo S, Fernández-López R, Ruiz R, Vielva L, de Toro M (2020). Pathways for horizontal gene transfer in bacteria revealed by a global map of their plasmids. Nat Commun.

[46] Ummels R, Abdallah AM, Kuiper V, Aâjoud A, Sparrius M (2014). Identification of a novel conjugative plasmid in mycobacteria that requires both type IV and type VII secretion. mBio.

[47] Ghinet MG, Bordeleau E, Beaudin J, Brzezinski R, Roy S (2011). Uncovering the prevalence and diversity of integrating conjugative elements in actinobacteria. PLoS One.

[48] Li X, Xie Y, Liu M, Tai C, Sun J (2018). oriTfinder: a web-based tool for the identification of origin of transfers in DNA sequences of bacterial mobile genetic elements. Nucleic Acids Res.

[49] Katoh K, Standley DM (2013). MAFFT multiple sequence alignment software version 7: improvements in performance and usability. Mol Biol Evol.

[50] Guindon S, Dufayard JF, Lefort V, Anisimova M, Hordijk W (2010). New algorithms and methods to estimate maximum-likelihood phylogenies: assessing the performance of PhyML 3.0. Syst Biol.

[51] Rognes T, Flouri T, Nichols B, Quince C, Mahé F (2016). VSEARCH: a versatile open source tool for metagenomics. PeerJ.

[52] Hatfull GF, Jacobs-Sera D, Lawrence JG, Pope W, Russell DA (2010). Comparative genomic analysis of 60 mycobacteriophage genomes: genome clustering, gene acquisition, and gene size. J Mol Biol.

[53] Lanza VF, Baquero F, de la Cruz F, Coque TM (2017). AcCNET (accessory genome constellation network): comparative genomics software for accessory genome analysis using bipartite networks. Bioinformatics.

[54] Shannon P, Markiel A, Ozier O, Baliga NS, Wang JT (2003). Cytoscape: a software environment for integrated models of biomolecular interaction networks. Genome Res.

[55] Sela I, Ashkenazy H, Katoh K, Pupko T (2015). GUIDANCE2: accurate detection of unreliable alignment regions accounting for the uncertainty of multiple parameters. Nucleic Acids Res..

[56] Letunic I, Bork P (2016). Interactive tree of life (iTOL) V3: an online tool for the display and annotation of phylogenetic and other trees. Nucleic Acids Res.

[57] RStudio Team (2015). RStudio: Integrated Development for R. RStudio.

[58] Fedrizzi T, Meehan CJ, Grottola A, Giacobazzi E, Fregni Serpini G (2017). Genomic characterization of nontuberculous mycobacteria. Sci Rep.

[59] Leão SC, Matsumoto CK, Carneiro A, Ramos RT, Nogueira CL (2013). The detection and sequencing of a broad-host-range conjugative IncP-1β plasmid in an epidemic strain of *Mycobacterium abscessus* subsp. bolletii [published correction appears in PLoS One. 2013;8(9).10.1371/annotation/5dd55ed1-2fb6-4672-9142-fb01331567e1]. PLoS One.

[60] Uchiya K, Takahashi H, Nakagawa T, Yagi T, Moriyama M (2015). Characterization of a novel plasmid, pMAH135, from *Mycobacterium avium* subsp. hominissuis. PLoS One..

[61] Lee H, Kim BJ, Kim BR, Kook YH, Kim BJ (2015). The development of a novel *Mycobacterium-Escherichia col*i shuttle vector system using pMyong2, a linear plasmid from *Mycobacterium yongonense* DSM 45126T. PLoS One..

[62] Seniya SP, Yadav P, Jain V (2020). Construction of *E. coli-Mycobacterium* shuttle vectors with a variety of expression systems and polypeptide tags for gene expression in mycobacteria. PLoS One..

[63] Smillie C, Garcillán-Barcia MP, Francia MV, Rocha EP, de la Cruz F (2010). Mobility of plasmids. Microbiol Mol Biol Rev.

[64] Thoma L, Muth G (2015). The conjugative DNA-transfer apparatus of Streptomyces. Int J Med Microbiol.

[65] Hochhut B, Marrero J, Waldor MK (2000). Mobilization of plasmids and chromosomal DNA mediated by the SXT element, a constin found in Vibrio cholerae O139. J Bacteriol.

[66] Douard G, Praud K, Cloeckaert A, Doublet B (2010). The Salmonella genomic island 1 is specifically mobilized in trans by the IncA/C multidrug resistance plasmid family. PLoS One.

[67] Lee CA, Thomas J, Grossman AD (2012). The Bacillus subtilis conjugative transposon ICEBs1 mobilizes plasmids lacking dedicated mobilization functions. J Bacteriol.

[68] Ramsay JP, Kwong SM, Murphy RJ, Eto KY, Price KJ (2016). An updated view of plasmid conjugation and mobilization in *Staphylococcus*. Mob Genet Elements..

[69] Guédon G, Libante V, Coluzzi C, Payot S, Leblond-Bourget N (2017). The obscure world of integrative and mobilizable elements, highly widespread elements that Pirate bacterial conjugative systems. Genes (Basel)..

[70] Garcillán-Barcia MP, Francia MV, de la Cruz F (2009). The diversity of conjugative relaxases and its application in plasmid classification. FEMS Microbiol Rev.

[71] Klümper U, Riber L, Dechesne A, Sannazzarro A, Hansen LH (2015). Broad host range plasmids can invade an unexpectedly diverse fraction of a soil bacterial community. Isme J..

[72] Guglielmini J, de la Cruz F, Rocha EP (2013). Evolution of conjugation and type IV secretion systems. Mol Biol Evol.

[73] Guzmán-Herrador DL, Llosa M (2019). The secret life of conjugative relaxases. Plasmid.

[74] Panda A, Drancourt M, Tuller T, Pontarotti P (2018). Genome-Wide analysis of horizontally acquired genes in the genus Mycobacterium. Sci Rep.

[75] Bordeleau E, Ghinet MG, Burrus V (2012). Diversity of integrating conjugative elements in actinobacteria: coexistence of two mechanistically different DNA-translocation systems. Mob Genet Elements.

[76] Liu M, Li X, Xie Y, Bi D, Sun J (2019). Iceberg 2.0: an updated database of bacterial integrative and conjugative elements. Nucleic Acids Res..

[77] Wozniak RA, Waldor MK (2009). A toxin-antitoxin system promotes the maintenance of an integrative conjugative element. PLoS Genet.

[78] Guglielmini J, Quintais L, Garcillán-Barcia MP, de la Cruz F, Rocha EP (2011). The repertoire of ice in prokaryotes underscores the unity, diversity, and ubiquity of conjugation. PLoS Genet..

[79] Hülter N, Ilhan J, Wein T, Kadibalban AS, Hammerschmidt K (2017). An evolutionary perspective on plasmid lifestyle modes. Curr Opin Microbiol.

[80] Cury J, Oliveira PH, de la Cruz F, Rocha EPC (2018). Host range and genetic plasticity explain the coexistence of integrative and extrachromosomal mobile genetic Elements [published correction appears in Mol Biol Evol. 2018 Nov 1;35(11):2850]. Mol Biol Evol..

[81] Pesesky MW, Tilley R, Beck DAC (2019). Mosaic plasmids are abundant and unevenly distributed across prokaryotic taxa. Plasmid.

[82] diCenzo G, Milunovic B, Cheng J, Finan TM (2013). The tRNAarg gene and engA are essential genes on the 1.7-Mb pSymB megaplasmid of *Sinorhizobium meliloti* and were translocated together from the chromosome in an ancestral strain. J Bacteriol.

[83] Tran TT, Belahbib H, Bonnefoy V, Talla E (2015). A comprehensive tRNA genomic survey unravels the evolutionary history of tRNA arrays in prokaryotes. Genome Biol Evol.

[84] Morgado SM, Vicente ACP (2018). Beyond the limits: tRNA array units in *Mycobacterium* genomes. Front Microbiol.

[85] Williams KP (2002). Integration sites for genetic elements in prokaryotic tRNA and tmRNA genes: sublocation preference of integrase subfamilies. Nucleic Acids Res.

[86] Rozwandowicz M, Brouwer MSM, Fischer J, Wagenaar JA, Gonzalez-Zorn B (2018). Plasmids carrying antimicrobial resistance genes in *Enterobacteriaceae*. J Antimicrob Chemother.

[87] Koraimann G (2018). Spread and persistence of virulence and antibiotic resistance genes: a ride on the F plasmid conjugation module. EcoSal Plus.

[88] Lerminiaux NA, Cameron ADS (2019). Horizontal transfer of antibiotic resistance genes in clinical environments. Can J Microbiol.

[89] Graf FE, Palm M, Warringer J, Farewell A (2019). Inhibiting conjugation as a tool in the fight against antibiotic resistance. Drug Dev Res.

[90] Smalla K, Jechalke S, Top EM, Detection P (2015). Characterization, and ecology. Microbiol Spectr.

[91] Jia B, Raphenya AR, Alcock B, Waglechner N, Guo P (2017). Card 2017: expansion and model-centric curation of the comprehensive antibiotic resistance database. Nucleic Acids Res.

[92] Radhouani H, Silva N, Poeta P, Torres C, Correia S (2014). Potential impact of antimicrobial resistance in wildlife, environment and human health. Front Microbiol..

[93] Allen HK, Donato J, Wang HH, Cloud-Hansen KA, Davies J (2010). Call of the wild: antibiotic resistance genes in natural environments. Nat Rev Microbiol.

[94] Ly A, Liu J (2020). Mycobacterial virulence factors: surface-exposed lipids and secreted proteins. Int J Mol Sci.

[95] Tinaztepe E, Wei JR, Raynowska J, Portal-Celhay C, Thompson V (2016). Role of metal-dependent regulation of ESX-3 secretion in intracellular survival of Mycobacterium tuberculosis. Infect Immun.

[96] Bosserman RE, Champion PA (2017). Esx systems and the mycobacterial cell envelope: what’s the connection?. J Bacteriol.

[97] Fishbein S, van Wyk N, Warren RM, Sampson SL (2015). Phylogeny to function: PE/PPE protein evolution and impact on *Mycobacterium* tuberculosis pathogenicity. Mol Microbiol.

[98] Nakanaga K, Ogura Y, Toyoda A, Yoshida M, Fukano H (2018). Naturally occurring a loss of a giant plasmid from *Mycobacterium ulcerans* subsp. shinshuense makes it non-pathogenic. Sci Rep.

[99] Fan X, Xie L, Li W, Xie J (2014). Prophage-like elements present in *Mycobacterium* genomes. BMC Genomics.

[100] Sassi M, Gouret P, Chabrol O, Pontarotti P, Drancourt M (2014). Mycobacteriophage-drived diversification of *Mycobacterium abscessus*. Biol Direct..

[101] Phelippeau M, Asmar S, Osman DA, Sassi M, Robert C (2017). "*Mycobacterium massilipolynesiensis*" sp. nov., a rapidly-growing *Mycobacterium* of medical interest related to *Mycobacterium phlei*. Sci Rep.

[102] Bobay LM, Touchon M, Rocha EP (2014). Pervasive domestication of defective prophages by bacteria. Proc Natl Acad Sci U S A.

[103] Smith BT, Walker GC (1998). Mutagenesis and more: umuDC and the *Escherichia coli* SOS response. Genetics.

[104] Tom EF, Molineux IJ, Paff ML, Bull JJ (2018). Experimental evolution of UV resistance in a phage. PeerJ.

[105] Ma L, Green SI, Trautner BW, Ramig RF, Maresso AW (2018). Metals enhance the killing of bacteria by bacteriophage in human blood. Sci Rep.

[106] Janssen BD, Hayes CS (2012). The tmRNA ribosome-rescue system. Adv Protein Chem Struct Biol.

[107] Weinberg Z, Regulski EE, Hammond MC, Barrick JE, Yao Z (2008). The aptamer core of SAM-IV riboswitches mimics the ligand-binding site of SAM-I riboswitches. RNA.

[108] Mazauric MH, Licznar P, Prère MF, Canal I, Fayet O (2008). Apical loop-internal loop RNA pseudoknots: a new type of stimulator of -1 translational frameshifting in bacteria. J Biol Chem.

[109] Weinberg Z, Perreault J, Meyer MM, Breaker RR (2009). Exceptional structured noncoding RNAs revealed by bacterial metagenome analysis. Nature.

[110] Delesalle VA, Tanke NT, Vill AC, Krukonis GP (2016). Testing hypotheses for the presence of tRNA genes in mycobacteriophage genomes. Bacteriophage.

[111] Morgado S, Vicente AC (2019). Global *In-Silico* scenario of tRNA genes and their organization in virus genomes. Viruses.

[112] Colavecchio A, Cadieux B, Lo A, Goodridge LD (2017). Bacteriophages contribute to the spread of antibiotic resistance genes among foodborne pathogens of the *Enterobacteriaceae* family - A review. Front Microbiol.

[113] Wendling CC, Refardt D, Hall AR (2020). Fitness benefits to bacteria of carrying prophages and prophage-encoded antibiotic-resistance genes peak in different environments. Evolution.

[114] Quan S, Venter H, Dabbs ER (1997). Ribosylative inactivation of rifampin by *Mycobacterium smegmatis* is a principal contributor to its low susceptibility to this antibiotic. Antimicrob Agents Chemother.

[115] Baysarowich J, Koteva K, Hughes DW, Ejim L, Griffiths E (2008). Rifamycin antibiotic resistance by ADP-ribosylation: structure and diversity of Arr. Proc Natl Acad Sci U S A.

