# Supplementary material for: Comprehensive in silico survey of the Mycolicibacterium mobilome reveals an as yet underexplored diversity
Source: Microb Genom. 2021 Feb 23;7(3):mgen000533. doi: 10.1099/mgen.0.000533 (PMC8190616; doi:10.1099/mgen.0.000533)
Supplement: Supplementary material 2 [file mgen-7-0533-s002.pdf]

Supplementary Figures

Fig. S1. Hierarchical clustering based on gANI. The colours indicate similarity: grey, 0-69; blue, 70-96.4; red, 96.5-100. This analysis included all known *Mycobacteriaceae* plasmids.

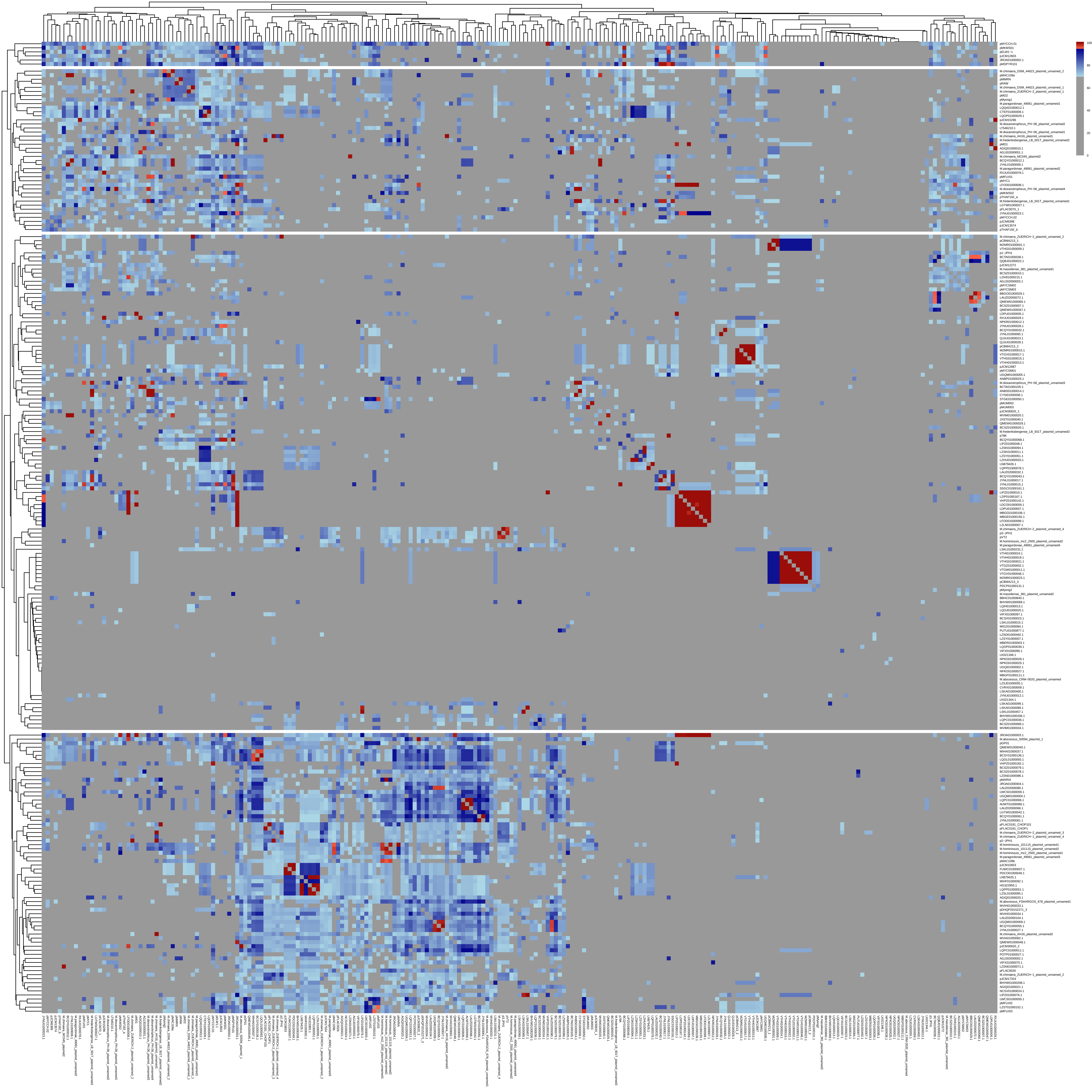

Fig. S2. Hierarchical clustering based on AF (red gradient). This analysis included all known *Mycobacteriaceae* plasmids.

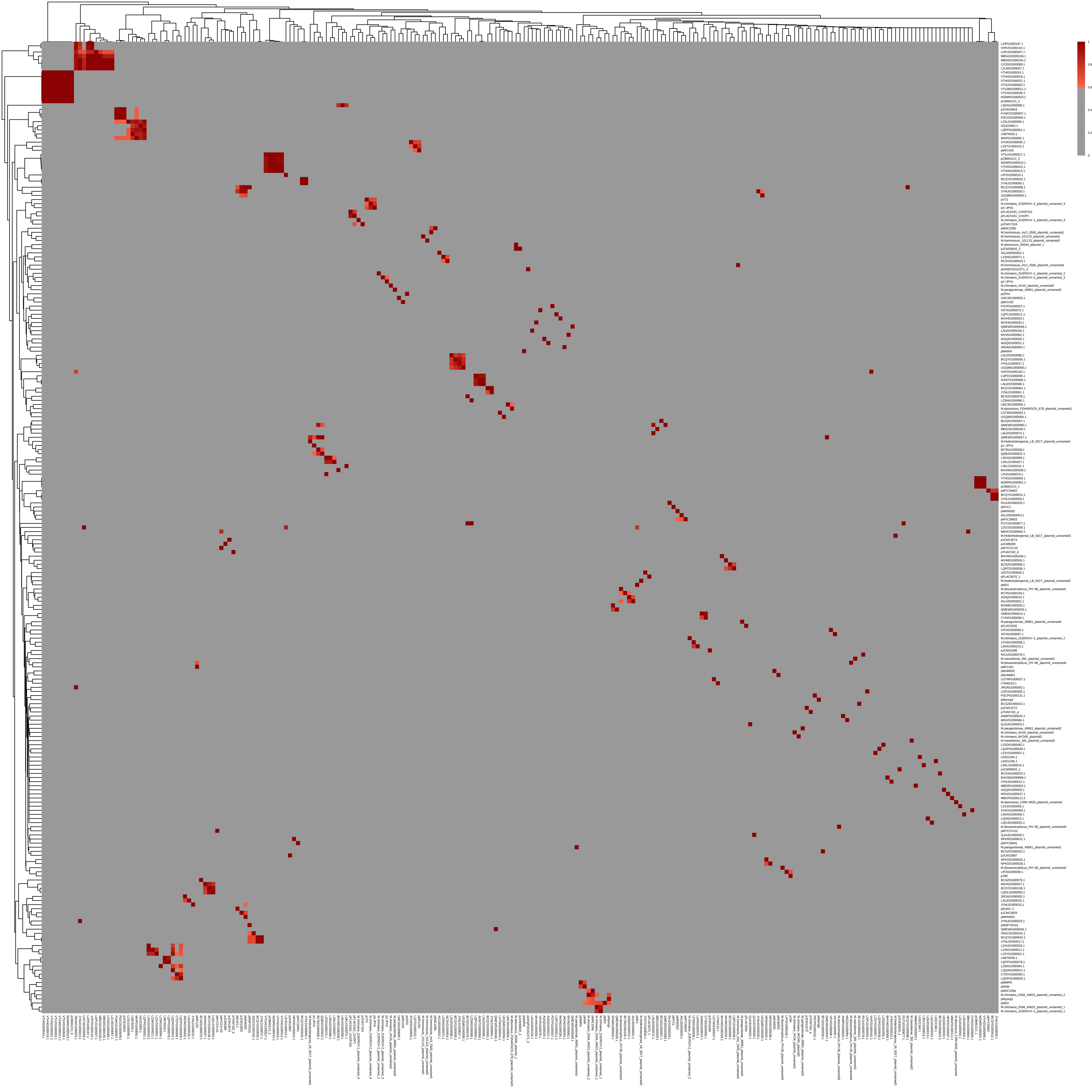

Fig. S3. Maximum-likelihood tree of the *rep* gene encoded by *Mycobacteriaceae* plasmids. The predicted plasmids are coloured in black, while the reference sequences of known *Mycobacteriaceae* plasmids are in red (*Mycobacterium*), blue (*Mycobacteroides*), and green (*Mycolicibacterium*). The green circles on the branches indicate bootstrap ≥ 90.

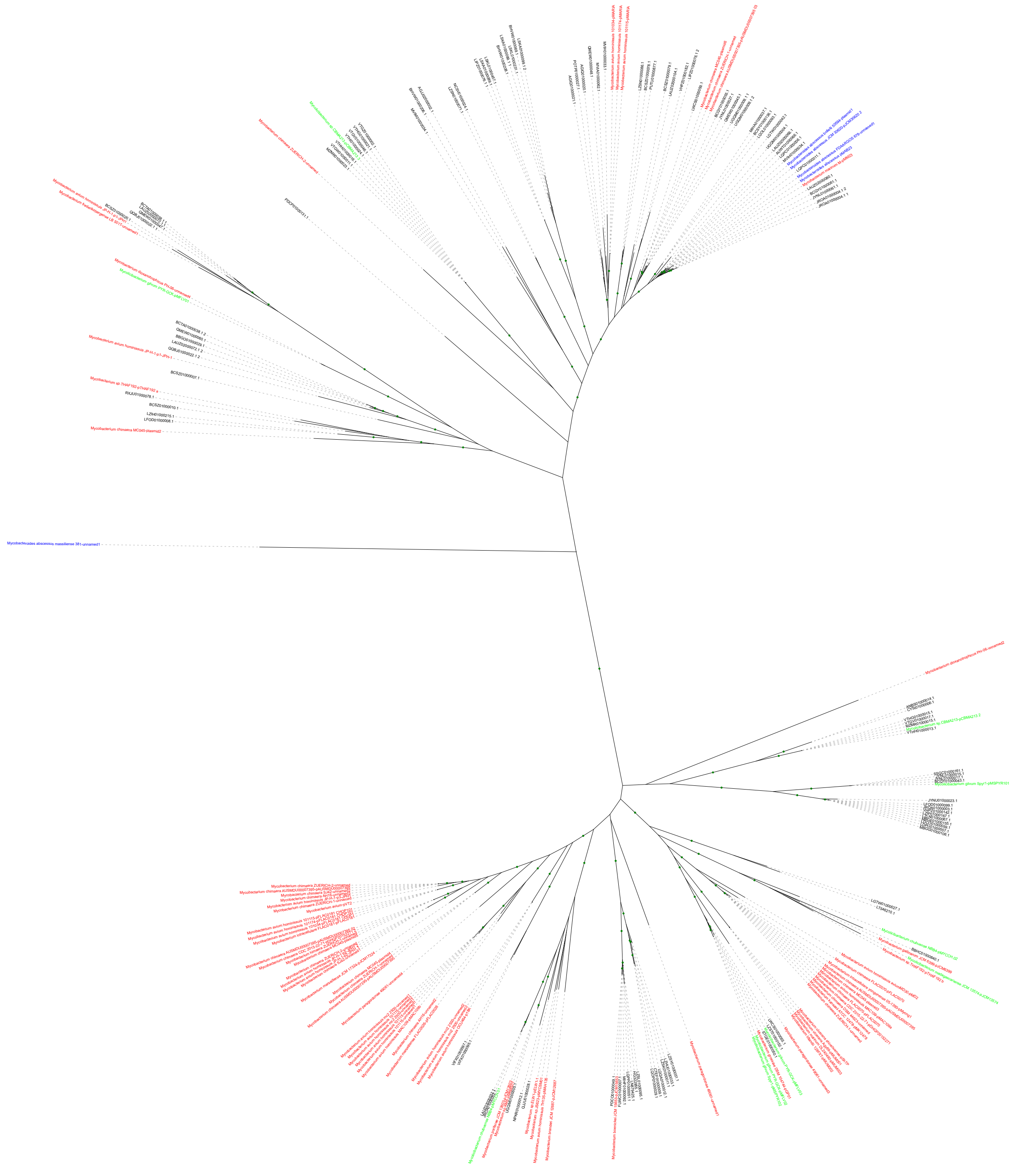

Mycobacteroides abscessus massiliense 381-unnamed1
Mycobacterium dioxanotrophicus PH-06-unnamed2
Mycobacterium chimaera MC045-plasmid2
Mycobacterium sp.THAF192-pTHAF192_a
Mycobacterium avium hominissuis JP-H-1-p1-JPH-1
